# Cobalt-engineered zeolitic imidazolate hexagonal nanocrystals as a robust and sustainable catalyst for hydrogen production *via* NaBH_4_ hydrolysis: experimental and theoretical validation

**DOI:** 10.1039/d5ra05946a

**Published:** 2025-09-24

**Authors:** Abhithrinayani Vedullacheruvu, Chob Singh, Rohan Magadum, Hemavathi Manjunath, Ashutosh Pandey, Akshaya K. Samal, Arvind H. Jadhav

**Affiliations:** a Centre for Nano and Material Sciences, Jain University Jain Global Campus Bangalore 562112 Karnataka India j.arvind@jainuniversity.ac.in jadhav.ah@gmail.com

## Abstract

The global economic growth mainly depends on energy consumption, with fossil fuels being a major contributor to environmental concerns. Hydrogen energy can be a greener replacement for future energy requirements. Herein, Co@ZIF was used as an efficient catalyst for hydrogen generation *via* NaBH_4_ hydrolysis. A facile strategy assisted the synthesis of Co@ZIF-*X* materials through the wet chemical route with reaction times of 60, 90, 120, 150, and 180 min for *X* = 1, 2, 3, 4, and 5, respectively. The Co@ZIF-5 catalyst showed superior catalytic activity for hydrogen generation *via* the hydrolysis of NaBH_4_ under ideal reaction conditions. This catalyst was optimized by studying the effect of catalyst amount, reaction time, temperature, and NaBH_4_ amount. Co@ZIF-5 catalyst demonstrated a high catalytic activity towards the hydrogen evolution reaction and produced 2701 mL of hydrogen w.r.t. a rateof 1350.5 mL min^−1^ g^−1^ in 80 min at room temperature. Interestingly, Co@ZIF-5 achieved efficient catalytic activity at a low activation energy of 14.95 kJ mol^−1^. Furthermore, the Co@ZIF-5 catalyst could be successfully recycled for up to 5 consecutive cycles without significant loss of activity. Theoretical calculations were also performed to assess hydrolysis results and crystal structure validation. This protocol demonstrated that Co@ZIF is a promising catalyst for hydrogen generation under green and sustainable conditions.

## Abstract 2

The hydrolysis of NaBH_4_ using an efficient Co@ZIF catalyst for H_2_ generation.

## Graphical abstract

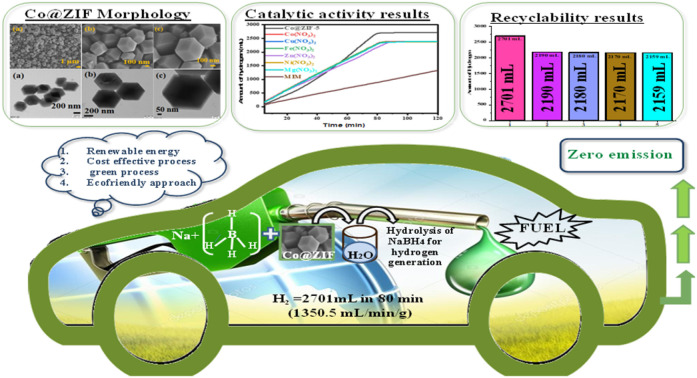

## 1. Introduction

The world's energy resources and fossil fuels are diminishing day by day. The use of non-renewable resources has led to the high consumption and consequent depletion of oil resources.^1,2^ Additionally, climate change around the world results in high environmental pollution. Traditional non-green energy resources such as fossil fuels produce toxic gases that promote global warming.^3^ Thus, scientific research has encouraged the use of alternative environmentally friendly energy resources.^4–6^ Hydrogen gas has emerged as an alternative sustainable zero-emission fuel with high energy content and environmentally friendly byproducts. Hydrogen possesses high energy density, cleanliness, and eco-friendliness. Although there are many techniques for hydrogen production, it is stored as high-pressure gas in the form of liquid hydrogen.^7^ In this case, the direct storage and transportation of hydrogen is a complex process. Considering its natural occurrence, it is the most abundant element in the universe. The sun and other stars are mainly composed of hydrogen. Astronomers estimate that 90% of the atoms in the universe are hydrogen atoms. Herein, we focus on the hydrolysis of NaBH_4_ because the splitting of water in the presence of hydrides generates hydrogen efficiently at low cost and without any emission of greenhouse gases. Water is the most abundant compound of hydrogen found on Earth. Due to the difficulty in developing hydrogen storage systems, the *in situ* hydrolysis reaction to produce hydrogen is a green and eco-friendly approach.

Twenty years ago, sodium borohydride (NaBH_4_) emerged as a desirable hydrogen carrier. Since the discoveries made by Schlesinger and colleagues during World War II, no new and fundamental insights have been found. However, a new window of opportunity for a carrier that can easily produce H_2_ in ambient surroundings has been created by current energy research. Sodium borate has a number of benefits. Its four hydrides, H^*δ*−^, react easily with the protic hydrogen of water, H^*δ*+^. The reaction is exothermic (Δ*H* = 210–270 kJ mol^−1^) and proceeds according to the standard path during hydrolysis.^8^

1 NaBH_4_ + 4H_2_O → NaB(OH)_4_ + 4H_2_

Remarkably, half of the four molecules of H_2_ are produced by water. A catalyst can speed up the release of H_2_, and the predicted gravimetric hydrogen storage capacity of the NaBH_4_–H_2_O couple is as high as 7.3 wt% based on the stoichiometry indicated by eqn (1). Sodium tetrahydroxyborate NaB(OH)_4_ (sometimes represented as NaBO_2_·2H_2_O), a water-soluble chemical with a solubility of 3.5 mol L^−1^, is the only by-product generated during hydrolysis.^9,10^ Relatively pure H_2_ is emitted, and the exothermicity of the reaction causes a few borates to be produced in the steam at the same time. Some disadvantages of sodium borohydride include the precipitation of borates, which can clog pipes, and the exothermic nature of the reaction, which necessitates heat management. Depending on how the hydride is treated, there are three different ways to construct the NaBH_4_ hydrolysis-based H_2_ generation process. The most researched design makes use of an alkaline NaBH_4_ aqueous solution. Contact between the solution and a heterogeneous metal-based catalyst initiates the hydrolysis reaction. At the laboratory scale, the catalyst is added to a flask containing NaBH_4_ and water. To maximize the amount of hydrogen produced and achieve rapid reaction rates, the hydride and catalyst should be closely combined. The hydrolysis process using hydrides is more effective because of the pure and regulated hydrogen produced. Metal hydrides are utilized for hydrolysis to produce hydrogen because of their high volumetric H_2_ storage density. This method has been applied to simple metal hydrides such as LiH, MgH_2_, CaH_2_, and AlH_3_, or complex metal hydrides such as LiBH_4_ and NaBH_4_ (ref. 11) in aqueous media, given that sodium borohydride (NaBH_4_) is a desirable reducing agent. Its high calorific value and gravimetric hydrogen density make it a popular material for hydrogen generation. However, a catalyst is required to accelerate the kinetically slow process.

As a result, a variety of materials have been employed as catalysts to produce hydrogen through the hydrolysis of NaBH_4_. Precious metal catalysts, such as Pt and Pd, are expensive, which restricts their use as catalysts.^12^ Alternatively, transition metals are inexpensive and as effective as the above-mentioned catalysts. A support is necessary when using transition metals to improve their performance and stop the catalytic species from aggregating. Compared to supports such as MXenes, MoS_2_, and carbon nanotubes (CNTs), ZIFs are better in supporting transition metals.^13^ The hydrolysis reaction is often exothermic and always takes place without the aid of a catalyst. Only when the NaBH_4_ solution comes into contact with certain appropriate catalysts can the hydrolysis of NaBH_4_ quickly release hydrogen. Accordingly, a variety of catalysts, including Pt, Rh, Ru, Ni, and Co, has been employed in recent decades to catalyse the hydrolysis of NaBH_4_.^14^ Among them, Co-based catalysts show the greatest potential because of their increased catalytic activity and lower cost, according to experimental data. Cobalt-based catalysts can be synthesized and modified using a variety of research techniques. In particular, a number of assisted catalysts have been investigated. Regarding hydrolyzing aqueous NaBH_4_, a cobalt-integrated ZIF catalyst synthesized using a wet chemical technique is more active than the unsupported catalyst. Because of its large specific surface area, the amorphous catalyst has great catalytic activity. As the support, Li *et al.* selected Co-ZIF-9 and discovered that while the reaction rate of Co supported on MOFs was excellent, the exothermic character of this reaction leads to the agglomeration of CoB particles.^9^ The effective surface area of the catalyst powder is reduced by this particle agglomeration, which restricts its catalytic activity. Therefore, to increase its catalytic activity and lower its production costs, new catalysts with greater dispersion of CoB must be produced. Metal organic frameworks (MOFs) are a new class of porous materials that has recently been developed.

In particular, the family of zeolitic imidazolate frameworks (ZIFs) has produced some highly active ZIF materials, which have been employed as catalysts in some common processes. With their hybrid frameworks made up of inorganic metal ions or metal clusters synchronized with organic imidazole or imidazolate ligands, ZIFs exhibit ordered porous architectures. ZIFs possess numerous appealing qualities, especially a high specific surface area and chemically flexible structure. Their distinct qualities make them attractive for further use, such as gas storage, separation, catalysis, and sensing.^11^ In the past 10 years, MOF-based hybrid materials, such zeolite imidazole frameworks (ZIFs), have been widely used because of their exceptional stability in water and their shape. Kanchanakanho *et al.* and Luo *et al.* provided significant insights into the catalytic performance of Co-based ZIF catalysts for NaBH_4_ hydrolysis. Kanchanakanho's group achieved the hydrogen generation of 720.7 mL min^−1^ g^−1^ with the activation energy of 63.74 kJ mol^−1^ using a cobalt-ZIF catalyst.^15^ Luo *et al.* group used a Co-ZIF-8 catalyst for the hydrolysis of NaBH_4_ and achieved 14 023 mL min^−1^ g^−1^ of H_2_ generation with an activation energy of 62.9 kJ mol^−1^.^16^ However, although these catalysts generated a high hydrogen rate, they showed a high activation energy, which makes the reaction more exothermic. Lu *et al.* synthesized a durable Ni–Co bimetallic catalyst supported by ZIF-67/rGO, which generated 5420 mL min^−1^ g^−1^ hydrogen with an activation energy of 21.08 kJ mol^−1^.^5^ Andrew *et al.* used a catalyst named magnetic cobalt/carbon composite derived from zeolitic imidazole frameworks and generated hydrogen with activation energy of 25.8 kJ mol^−1^.^17^ Metal-imidazole and hydrophobic linkages endow ZIF with its unique porosity structure, selectivity, and exceptional thermal and chemical stability.^18^ ZIFs have several interesting qualities, such as chemically flexible frameworks and high specific surface area, and therefore ZIFs have a positive perspective in catalysis.^19^

In the present study, Co@ZIF catalysts were synthesized by a wet chemical method without using a support substrate such as MXene and rGO, and used to efficiently generate hydrogen *via* the hydrolysis of NaBH_4_. Many previous studies have shown the hydrolysis of NaBH_4_, and some reports in the literature even achieved a high H_2_ generation rate but they lacked a low activation energy. The as-synthesized Co@ZIF catalyst exhibited a low activation energy (*E*_a_), which is much better than the previously reported catalysts. This work focuses on a cost-effective and short synthesis process and targets the hydrolysis of NaBH_4_ for hydrogen generation with a low activation energy, which can be utilized to alleviate climate change and achieve a green energy economy.

## 2. Materials and methods

### 2.1. Materials

2-Methylimidazole (2-MIM) (99%), cobalt nitrate hexahydrate (Co(NO_3_)·6H_2_O) (99%) and methanol (MeOH, 99.5%), sodium borohydride (NaBH_4_, 98%), and distilled water. MIM, cobalt nitrate hexahydrate, and NaBH_4_ were purchased from Sigma-Aldrich, India. Methanol was purchased from Merck. All the obtained chemicals were used without further purification. Distilled water (laboratory grade, produced in-lab using a Milli-Q purification system) was produced in our laboratory with a conductivity less than 10^−4^ S m^−1^.

### 2.2. General synthesis of Co@ZIF nanocrystals *via* wet chemical method

**Scheme 1:**
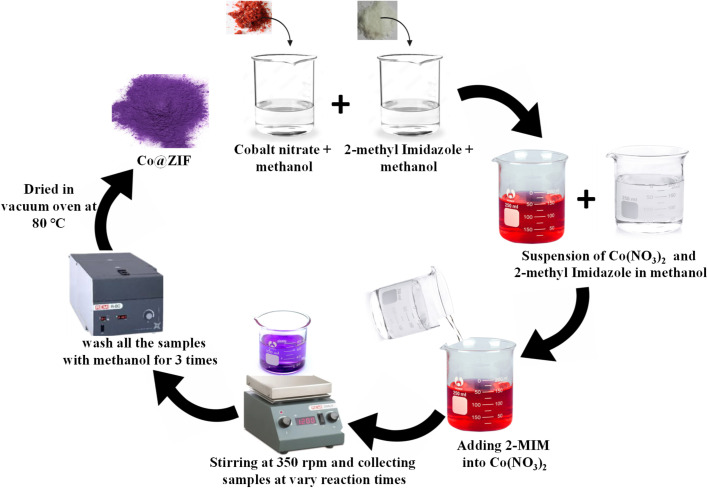
General schematic representation of the synthesis of Co@ZIF catalysts.

The wet chemical method was followed to synthesize the Co@ZIF nanocrystals. In this method, 1.1 g of 2-methyl imidazole was dissolved in 50 mL of methanol, and 1 g of cobalt salt (cobalt nitrate hexahydrate) was separately mixed in 50 mL of methanol. The former solution was poured into the latter and stirred at a constant speed of 350 rpm with varying reaction times of 60, 90, 120, 150, and 180 min. The resultant precipitates were collected and washed with methanol thrice and dried in a vacuum oven overnight at 80 °C. A schematic representation of the synthesis process and sample details is given below in Scheme 1 and Table 1. The obtained samples were designated as Co@ZIF-1, Co@ZIF-2 Co@ZIF-3, Co@ZIF-4 and Co@ZIF-5, respectively. The above-synthesized Co@ZIF catalysts were used to generate hydrogen *via* the hydrolysis of NaBH_4_.

**Table 1:** Experimental conditions for various synthesized ZIF crystals. The most efficient sample is highlighted in bold

Trail no.	Sample	2-MIM : Co(NO_3_)_2_·6H_2_O (g)	Reaction time (min)
1	Co@ZIF-1	1.10 : 1	60
2	Co@ZIF-2	1.10 : 1	90
3	Co@ZIF-3	1.10 : 1	120
4	Co@ZIF-4	1.10 : 1	150
**5**	**Co@ZIF-5**	**1.10 : 1**	**180**

### 2.3. General hydrolysis process

**Scheme 2:**
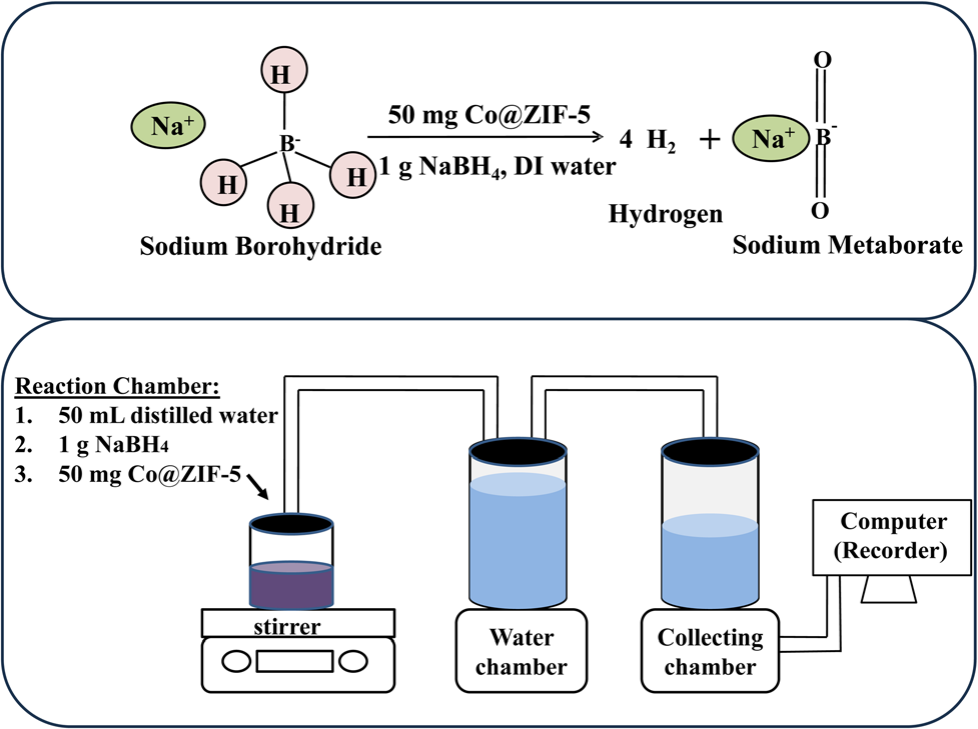
Schematic of the hydrolysis reaction setup.

Employing the hydrolysis reaction setup, as shown in Scheme 2, the water displacement method was used to generate hydrogen from the hydrolysis of NaBH_4_. To employ the hydrolysis reaction by NaBH_4_, we used 1 g of NaBH_4_ and 50 mg of Co@ZIF catalyst and 50 mL volume of distilled water to generate hydrogen. A magnetic stirrer was attached to the reaction chamber, which was connected by a pipe. A pipe that was attached to the water chamber carried the hydrogen gas that was produced in the reaction chamber. An additional pipe was taken out of the water chamber, connected to the collecting chamber that was empty, and set up on an electronic balance. The hydrogen gas generated in the reaction chamber traveled through the pipe and created pressure in the water chamber during the reaction path. The values were then recorded regarding time after an equivalent volume of water was displaced in the collecting chamber, which was maintained on the electronic balance. As a result, the amount of hydrogen generated during hydrolysis was demonstrated by the released H_2_ gas from the reaction chamber dislodging an equivalent volume of water in the collecting chamber. The magnetically active catalyst was extracted from the reaction mixture using an external magnetic bar once the reaction was finished. After washing 3–4 times with water, and then ethanol, the separated catalyst was restored and dried in a hot air oven for 12 h at 80 °C. This was then utilized for additional cycles in the hydrolysis process.^20^ A systematic study on the hydrolysis of NaBH_4_ was carried out in terms of the effect of reaction time, temperature, and NaOH, HCl, and NaBH_4_ concentration on the catalytic reaction.

### 2.4. Environmental considerations and waste management of current methodology

One of the promising and eco-friendly ways to produce hydrogen is *via* the hydrolysis of NaBH_4_ with Co@ZIF catalysts. However, its long-term viability depends on a thorough assessment of its environmental impact. Sodium metaborate (NaBO_2_), the by-product, builds up over various reaction cycles and can cause environmental contagion and problems with waste disposal. This problem might be resolved by chemically regenerating NaBO_2_ back to NaBH_4_, which would eliminate the need for new reagents. Recent research has investigated chemical reduction and electrochemical methods for NaBO_2_ regeneration. The energy efficiency during catalyst regeneration is a concern due to the exothermic nature of the NaBH_4_ hydrolysis reaction. The environmental performance of the process could be enhanced by low energy drying techniques or alternative catalyst recovery methods. Also, the solvents, especially methanol, must be handled and disposed of safely.^21^ Given that methanol is a volatile organic compound and poses environmental and health risks, its proper containment, recycling, or substitution with greener solvents should be considered. Following green chemistry principles such as solvent recovery, minimizing hazardous waste, and employing sustainable synthesis routes can ensure that the overall process remains eco-friendly.^22^

To minimize the risk of overheating and related safety issues, the reaction was carried out under tightly controlled conditions, with continuous monitoring and effective heat dissipation strategies in place. All temperature-sensitive experiments were performed within a fume hood to maintain a safe working environment. The aim of this study was not to complete a comprehensive investigation of temperature-dependent behaviour, but rather to gain initial insights into how temperature influences the hydrolysis of NaBH_4_. These precautions ensured safe experimental operation and helped avoid any negative outcomes.

Furthermore, to enhance the life cycle sustainability of the Co@ZIF catalyst, future studies should investigate its long-term stability over multiple reaction cycles. Understanding how catalyst deactivation arises and developing strategies to prevent performance degradation such as surface variation and protective coatings can improve the catalyst durability, minimize material wastage and reduce the frequency of catalyst replacement. By addressing these environmental considerations such as by-product recycling, energy efficiency, solvent management, and catalyst durability, this hydrogen generation process can be further optimized to align with the principles of sustainable and green energy technologies.

### 2.5. General methods for catalyst characterization

Numerous analytical and spectroscopic characterization investigations were performed to investigate the physicochemical properties of the as-synthesized catalysts. A JEOL field emission scanning electron microscope (JSM-7000F, Singapore) equipped with energy-dispersive X-ray (EDX) spectroscopy was used to identify the as-synthesized catalysts. The polycrystalline characteristics of the synthesized product were investigated using powder X-ray diffraction (*p*-XRD) (Rigaku X-ray diffraction, Japan) at a scan rate of 2° min^−1^. The XRD patterns of the produced materials were analyzed in the 2*θ* range of 5° to 80°. Additionally, the functional groups present in the Co@ZIF nanocrystals were investigated using a Fourier transform infrared (FT-IR) spectrometer (PerkinElmer, Spectrum 400). Raman analysis was carried out to analyse the Raman active modes, and the sharp intense peaks confirm the chemical structure and crystal nature of the Co@ZIF catalyst. Thermogravimetric analysis (TGA) was performed for the synthesized Co@ZIF-5 material to check its thermal stability. For the further confirmation of the morphological analysis, transmission electron microscopy (TEM) and high-resolution transmission electron microscopy (HR-TEM) (JEOL (Japan)) were conducted for the Co@ZIF catalyst. The zeta potential of the synthesized nanoparticles was measured using a Litesizer TM 500 from Anton Paar. X-ray photo electron spectroscopy (XPS) analysis was carried out to confirm the oxidation state of the Co@ZIF-5 catalyst.

## 3. Results and discussion

Zeolitic imidazolate frameworks (ZIFs), a subclass of metal–organic frameworks (MOFs), are known for their exceptional chemical and thermal stability, particularly in aqueous environments. Their importance in hydrolysis systems from both their resilience to hydrolysis and their utility as catalysts or supports in hydrolysis reactions. In this work, a cobalt-embedded ZIF was synthesized. We synthesized different types of Co@ZIF nanocrystals based on the reaction time, as mentioned in Table 1. All the synthesized Co@ZIFs were characterized using various spectroscopic and analytical techniques such as FE-SEM, EDAX, XRD, FT-IR, Raman, TGA, TEM & HR-TEM, and XPS, and also all the analyses were discussed in depth.

### 3.1. FE-SEM and EDAX analysis of synthesized Co@ZIF catalysts

**Fig. 1:**
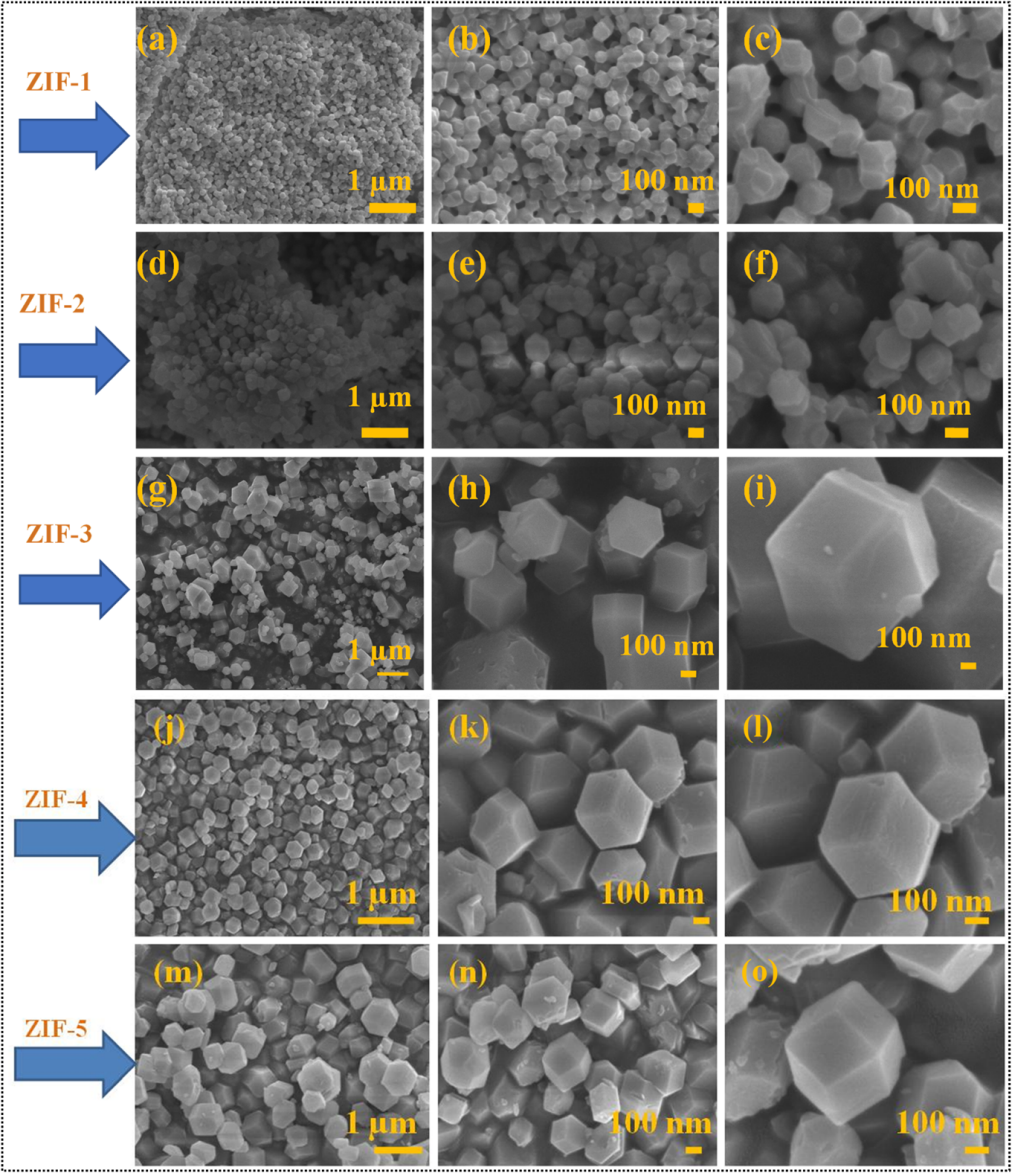
FE-SEM images: (a–c) Co@ZIF-1, (d–f) Co@ZIF-2, (g–i) Co@ZIF-3, (j–l) Co@ZIF-4, and (m–o) Co@ZIF-5 hexagonal crystals.

FE-SEM analysis was conducted to investigate the surface morphology of the synthesized Co@ZIF samples. All the results obtained from the FE-SEM analysis are displayed in Fig. 1. Highly crystalline Co@ZIF crystals with well-defined hexagonal shapes were observed regardless of the synthesis time. The morphological characteristic of Co@ZIF (zeolite imidazole frameworks) at various reaction times were analyzed using FE-SEM at the magnifications of 1 μm and 100 μm, as shown below. The FE-SEM images of the Co@ZIF catalysts are shown in Fig. 1. These images show the surface morphology of Co@ZIF-1, 2, 3, 4, 5, which showed a hexagonal nanocrystal morphology, playing a vigorous role in their catalytic activity. The FE-SEM images of all the synthesized Co@ZIF-1, 2, 3, 4, 5 hexagonal nanocrystals are presented in Fig. 1(a–c), (d–f), (g–i), (j–l), and (m–o), respectively. Here, the ZIF catalyst, which was synthesized at the reaction time of 180 min and named Co@ZIF-5, is considered it the most promising catalyst for hydrogen generation.^20^ The obtained FE-SEM images of the as-synthesized Co@ZIF-5 catalyst are shown in Fig. 1(m–o), which showed hexagonal nanocrystals without agglomeration.

Besides, by increasing this reaction time, the metal ions further form bonds between the imidazole rings and help in growth of hexagonal nanocrystals. Alternatively, massive hexagonal nanocrystals are produced when nuclei grow comparatively at ambient temperature. As a result, the size and shape of the Co@ZIF-5 crystals tend to be more consistent and regular in structure under the reaction conditions (such as constant stirring at ambient temperature). These well-defined hexagonal nanocrystals suggest high crystallinity and potentially large surface areas. These higher surface areas facilitate Co dispersion and more accessible active sites, improving hydrogen generation.^23^ The reaction time played an important role in determining the morphological aspects of the synthesized ZIFs. At shorter reaction times such as 60, 90, and 120 min, the catalysts predominantly consisted of smaller and irregularly shaped particles, which can be attributed to the incomplete nucleation and limited crystal growth for Co@ZIF-1, Co@ZIF-2, and Co@ZIF-3, respectively. As the reaction time increased as 150 and 180 min, the particles gradually transformed into more uniform and well-defined hexagonal structures, indicating the enhanced crystallinity and growth stability of the Co@ZIF-4 and Co@ZIF-5 catalysts. Prolonged reaction times further promoted the development of larger, highly ordered crystals with smoother surfaces. These observations clearly suggest that extended reaction durations facilitate sufficient nucleation and growth, thereby improving both the morphology and overall structural integrity of the ZIFs.

**Fig. 2:**
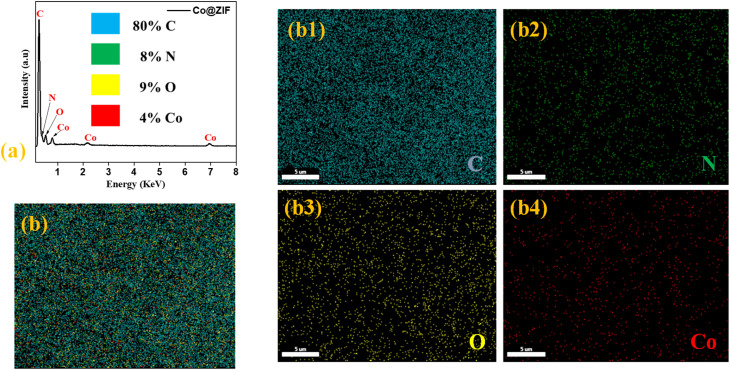
(a) EDX analysis of Co@ZIF-5, (b) elemental composition of Co@ZIF-5 nanocrystals, and (b1–b4) elemental mapping of carbon, nitrogen, oxygen, and cobalt.

Together with FE-SEM and elemental mapping, EDX analysis is used to ascertain the elements present in the prepared materials. Fig. 2 shows the results obtained for Co@ZIF-5. The Co, C, N, and O elements were found in the EDX analysis of the synthesized Co@ZIF-5. Co particles are responsible for the increased intensity of the new peaks. The findings demonstrated the hexagonal structure of the Co@ZIF nanocrystals. The sizes and crystal shape acquired at various reaction periods were consistent with the SEM results. Shortening the reaction period from 180 to 60 min resulted in modest degradation of sodalite shape, despite the hexagonal morphology and crystal size remaining intact. Furthermore, the initial nucleation also caused a modest decrease in the crystallinity of the crystals. As seen in Fig. 2(a) and (b) displays the EDX mapping of the Co@ZIF-5 nanocrystals with the good dispersion of the existing elements, and Fig. 2(b1–b4) illustrate the elemental distribution of C, N, O, and Co, respectively. This elemental mapping EDX was carried out in a specific area of the SEM micrograph to further observe the spatial distribution of Co@ZIF. Owing to the uniform distribution of all the elements, especially Co, they act as the active centres for hydrogen generation, likely facilitating the hydrolysis of NaBH_4_. The high surface area of the Co@ZIF-5 framework, combined with well dispersed Co, enhances the exposure of the active sites. The C and N elements contribute to the structural integrity and electron-donating environment, thus improving the Co catalytic efficiency. This synergy between the composition and structure enhances the catalytic behavior of Co@ZIF-5, making it effective even at room temperature.

### 3.2. FT-IR analysis of synthesized Co@ZIF catalysts

**Fig. 3:**
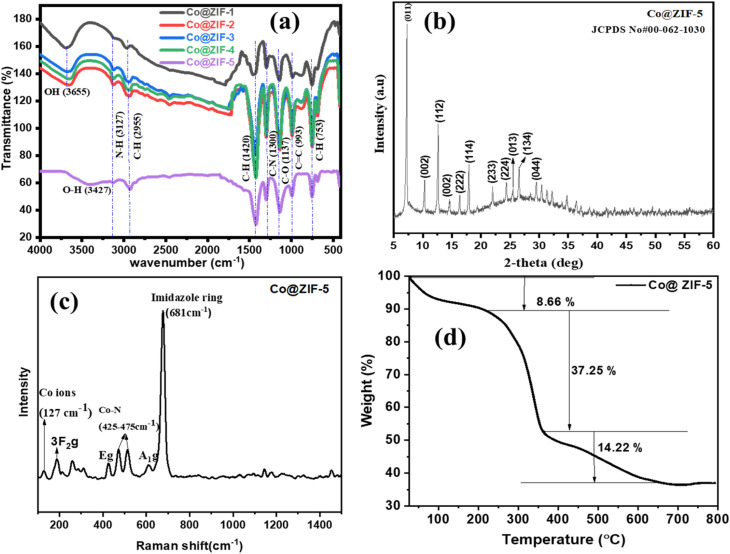
(a) FT-IR spectra of different Co@ZIF nanocrystals prepared at various reaction times, (b) XRD spectra of the Co@ZIF-5 catalyst, (c) Raman spectra, and (d) TGA of the Co@ZIF-5 catalyst.

Fig. 3(a) shows the FT-IR spectra of the Co@ZIF crystals prepared as supports at various times. The OH and N–H stretching vibrations are related to the peak at 3655 cm^−1^ for Co@ZIF, as shown in Fig. 3(a), while the N–H stretching vibration in MIM is related to the peak at 3127 cm^−1^. The aliphatic C–H stretching vibrations are associated with the band at around 2955 cm^−1^. Additionally, the C–H, C–N, and C–O bond: N stretching vibrations are represented by the peaks at 1421, 1300, and 1137 cm^−1^, respectively. Additionally, the peaks between 1250 and 1500 cm^−1^ are associated with the stretching of the imidazole ring, while the other peaks below 1000 cm^−1^ are associated with the in-plane and out-of-plane bending. Furthermore, the Co and N bond stretching vibration is attributed to the band at 753 cm^−1^, demonstrating the connection between MIM and Co^2+^. Consequently, the FT-IR spectrum of the Co@ZIF-5 catalyst displayed in Fig. 3(a) suggests that ZIF with cobalt essential peaks were added to the support.^24^ The FT-IR spectra of the synthesized Co@ZIF supports are displayed in Fig. 3(a). In these spectra, the band located at 424–647 cm^−1^ shows that the Co@ZIF structure remains stable during the impregnation of cobalt. Furthermore, OH stretching vibrations in MIM, which interact with Co and bond, and N stretching were identified as the source of the new bands between 2800 and 3200 cm^−1^ and the band located at 1454 cm^−1^ is associated with the stretching and bending vibrations of the N–H bond. The peaks located at 1455 and 777 cm^−1^ are associated with the C–H and N bonds, respectively. In comparison with Co@ZIF-*X* (*X* = 1, 2, 3, 4, 5), it shows the same functional groups at all the reaction times.^25,26^

### 3.3. XRD analysis of synthesized Co@ZIF-5 catalyst

X-ray diffraction analysis was performed to understand the crystalline phase and crystalline configuration of the Co@ZIF-5 catalyst, as shown in Fig. 3(b). All the XRD peaks matched with the crystal structure of CoH_7_C_3_NO_6_ (.cif file taken from https://next-gen.materialsproject.org/) generated using pymatgen, indicating a hexagonal structure with *P*6_3_ space group, which gave prominent peaks at 2*θ* values of 7.29°, 10.30°, 12.62°, 14.58°, 16.34°, 17.97°, 22.01°, 24.38°, 25.50°, and 26.52°, corresponding to the (110), (200), (211), (002), (013), (222), (114), (233), (224), and (134) crystal phases, respectively. These peaks reflect the high crystallinity and hexagonal symmetry of the framework.^27,28^ No additional peaks associated with the cobalt precursor and other impurities were observed, suggesting the high phase purity. The absence of diffraction signals from the precursors indicates that the cobalt was successfully incorporated into the framework without forming unwanted byproducts.^29,30^ The recorded XRD pattern of the synthesized Co@ZIF catalyst well matched JCPDS Card Number# 00-062-1030. Upon its application in hydrogen generation studies, the crystalline integrity of Co@ZIF-5 remained largely unchanged, indicating its good structural stability under hydrolysis conditions. The high crystallinity of Co@ZIF-5 suggests its well-defined structure and high surface area, which are favorable for the hydrogen evolution reaction. Furthermore, the XRD pattern shows no collapse of its structure upon Co incorporation, implying the thermal and chemical stability of this material, which are crucial for room temperature-dependent hydrogen generation.^31^ The structural and microstructural properties of the material were calculated and explained in a separate section.

### 3.4. Raman analysis of synthesized Co@ZIF-5 catalyst

Raman analysis was performed for the Co@ZIF catalyst to get structural evidence of the synthesized material. As shown in Fig. 3(c), sharp peaks were observed in the Raman spectrum at 127 cm^−1^, 187 cm^−1^, 425 cm^−1^, 474 cm^−1^, 514 cm^−1^, 610 cm^−1^, and 675 cm^−1^. The peak observed in the Raman spectrum at 127 cm^−1^ is attributed to the cobalt ions in Co@ZIF.^32^ The peaks at 425 cm^−1^ and 474 cm^−1^ indicate the presence of Co–N bonds in the catalyst. The peaks at 610 cm^−1^ and 675 cm^−1^ are associated with Co@ZIF, representing the N–H stretching and vibration mode of 2-methyl imidazole, which indicate C=N stretching (imidazole ring puckering).^18,33^ In these spectra, the Raman bands show symmetry at 127 cm^−1^, 187 cm^−1^, 260 cm^−1^, 425 cm^−1^, 474 cm^−1^, 514 cm^−1^, 610 cm^−1^, and 675 cm^−1^. At 127 cm^−1^ and 187 cm^−1^, the Raman bands exhibit ^3^F_2g_ symmetry. The peaks centered at 260 cm^−1^ correspond to E_g_ symmetry; however, the peaks located at 425 cm^−1^, 474 cm^−1^, 610 cm^−1^, and 675 cm^−1^ exhibit A_1g_ symmetry. The observed Raman active modes and sharp intense peaks confirm the chemical structure and crystal nature of the Co@ZIF catalyst.

Raman spectroscopy is sensitive to the hydride species that interact with the Co@ZIF framework, and thus the changes in the Raman spectra under catalytic conditions can indicate the formation of intermediates.^34^ The Co@ZIF catalyst undergoes structural transformation during the hydrogen generation process such as changes in the oxidation state of cobalt ions and their coordination environment.^30^ The hydrolysis reaction enhances the generation of protons and electrons at the active sites, followed by the formation of molecular hydrogen. Thus, the shifts in the Raman peaks suggest the interactions between the hydrogen and metal sites within the Co@ZIF framework.

### 3.5. TG analysis of synthesized Co@ZIF-5 catalyst

Thermo-gravimetric analysis (TGA) was carried out to check the thermal behavioral of the Co@ZIF-5 catalyst. The TGA thermogram showed a three-step decomposition with a total weight loss of 60.13% in the Co@ZIF-5 catalyst, as shown in Fig. 3(d). The first weight loss of 8.66% was ascribed to moisture loss in the precursor present in the catalyst. The second decomposition step in the temperature range of 221 °C to 363 °C with a weight loss of 37.25% is due to the formation of crystals. In the temperature range of 363 °C to 613 °C, a weight loss of 14.22% was observed, which is due to the change in the precursor to sodalite.^35^ A further increase in the temperature up to 800 °C did not display weight loss, which confirms the thermal stability of the as-synthesized Co@ZIF-5 catalyst.^36,37^ According to the TGA curves, the stable temperature of the catalyst was fixed to 613 °C. This confirms that Co@ZIF-5 obtained after 613 °C was highly stable. The highly stable catalyst strengthens the adsorption of protons (H^+^) for hydrogen generation in the hydrolysis reaction.^38^

### 3.6. TEM and HR-TEM analysis of synthesized Co@ZIF-5 catalyst

**Fig. 4:**
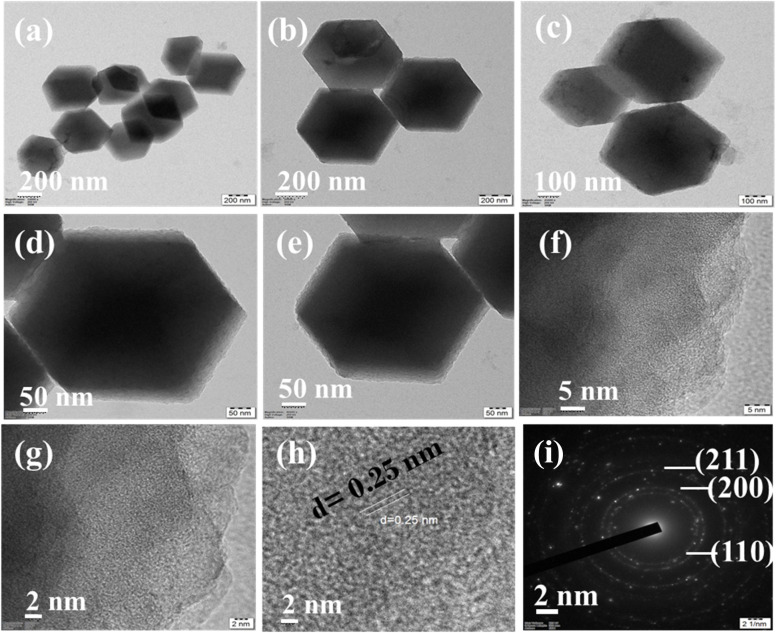
(a–e) TEM images of the synthesized Co@ZIF-5 catalyst, (f and g) HR-TEM images, (h) *d*-spacing between two consecutive lines, and (i) SAED pattern of the Co@ZIF-5 catalyst.

Transmission electron microscopy (TEM) and high-resolution transmission electron microscopy (HR-TEM) were used to further confirm the surface morphology of the produced Co@ZIF-5 nanocrystals. The TEM micrographs of the Co@ZIF-5 nanocrystals at different magnifications are shown in Fig. 4(a–e). The findings demonstrated the hexagonal structure of the Co@ZIF-5 nanocrystals. The size and crystal shape measured during the reaction were consistent with the FE-SEM results for the Co@ZIF-5 catalyst. In addition to its crystal size, there was a small degradation in the sodalite shape, even though the hexagonal morphology was preserved. Furthermore, the initial nucleation also caused a modest decrease in the crystallinity of the crystals.^39^Fig. 4(a–c) illustrate the low-magnification TEM images, showing uniformly dispersed, well defined hexagonal nanocrystals. The particle size distribution appears relatively constant, with lateral dimensions varying approximately from 100 nm to 200 nm, confirming the formation of nanocrystals. These hexagonal morphologies suggest a highly crystalline growth pattern, probably characteristic of the crystallographic orientation.^40^ The higher magnification images in Fig. 4(d and e) deliver a closer view of the individual particles, further showing the sharp edges and flat surface characteristic of the crystalline domains. The high-resolution TEM (HR-TEM) images shown in Fig. 4(f and g) revealed well-resolved lattice fringes, which confirm the crystalline nature of the catalyst. Fig. 4(h) shows the lattice fringes with an interplanar spacing (*d*-spacing) of 0.25 nm, which can be indexed to a specific crystal plane, likely corresponding to the plane of face centered cubic (FCC). The SAED pattern in Fig. 4(i) exhibits clear diffraction spots arranged in concentric rings indexed to the (110), (200), (211) planes. This confirms the phase purity and crystallinity. Here, the selected area electron diffraction (SAED) pattern also well matched with the *hkl* planes in the XRD pattern of Co@ZIF-5. The recorded SAED pattern of the Co@ZIF-5 catalyst is presented in Fig. 4(i).

### 3.7. XPS analysis of synthesized Co@ZIF-5 catalyst

**Fig. 5:**
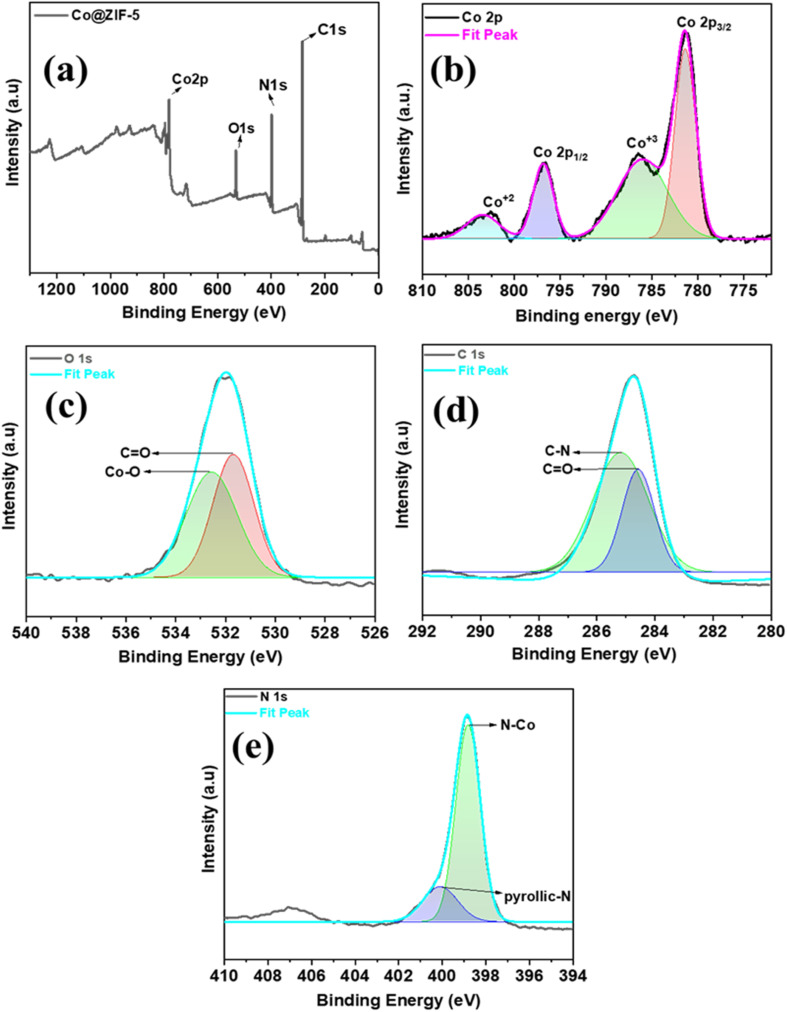
(a) High-resolution XPS profiles for the synthesized Co@ZIF-5 nanocrystals, (b) Co 2p spectra, (c) O 1s spectra, (d) C 1s spectra, and (e) N 1s spectra.

High-resolution X-ray photoelectron spectroscopy (XPS) was used to assess the valence electronic states and chemical composition of the optimized Co@ZIF-5 nanocrystals. The results are shown in Fig. 5(a–e). The comprehensive XPS survey scan, as shown in Fig. 5(a), indicates that the Co@ZIF-5 catalyst contains C, O, N, and Co components. The high-resolution spectrum of C 1s revealed peaks at the binding energies of 285.19 and 284.61 eV, corresponding to C–N and C=O, respectively, as shown in Fig. 5(d). Here, the C=O bonds are generally more helpful in hydrogen generation than the C–N bonds; their polar nature and ease of dehydrogenation make them better candidates in catalytic hydrogen generation.^41^ As seen in the high-resolution O 1s spectrum shown in Fig. 5(c), the peaks located at the binding energies of 531.69 and 532.50 eV were related to C=O and C–O, respectively. Among the peaks, the Co–O bonds are the most helpful in hydrogen generation by mediating electron transfer and stabilizing the reaction intermediates to improve the catalytic activity.^32^

Two peaks corresponding to pyrrolic-N (400.1 eV) and Co–N (399.4 eV) were visible in the high-resolution N 1s spectra (Fig. 5(e)). The Co 2p high-resolution XPS profile is further shown in Fig. 5(b), where the Co 2p_1/2_ and Co 2p_3/2_ states are identified by the peaks located at the binding energies of 796.8 eV and 781.38 eV, respectively. Consequently, Co 2p (2p_1/2_ and 2p_3/2_) had a spin–orbit splitting of 15.42 eV, demonstrating the oxidation state of Co^2+^. The satellite peaks located at the binding energies of 803.43 and 786.01 eV were associated with the Co^2+^ and Co^3+^ states, respectively. Between the above-mentioned states in cobalt, Co^2+^ helps to generate hydrogen generation efficiency compared to Co^3+^ under the typical conditions, especially in catalytic systems due to its greater stability in aqueous medium and more commonly involved in the hydrolysis reaction. Co^2+^ is reduced to Co^0^ easily, which is catalytically active for hydrogen generation, whereas Co^3+^ is more oxidized and less likely to directly participate in hydrogen generation.^42,43^

## 4. Catalytic activity

### 4.1. Determination of the catalytic activity of synthesized different Co@ZIF catalysts on hydrolysis rate

**Scheme 3:**
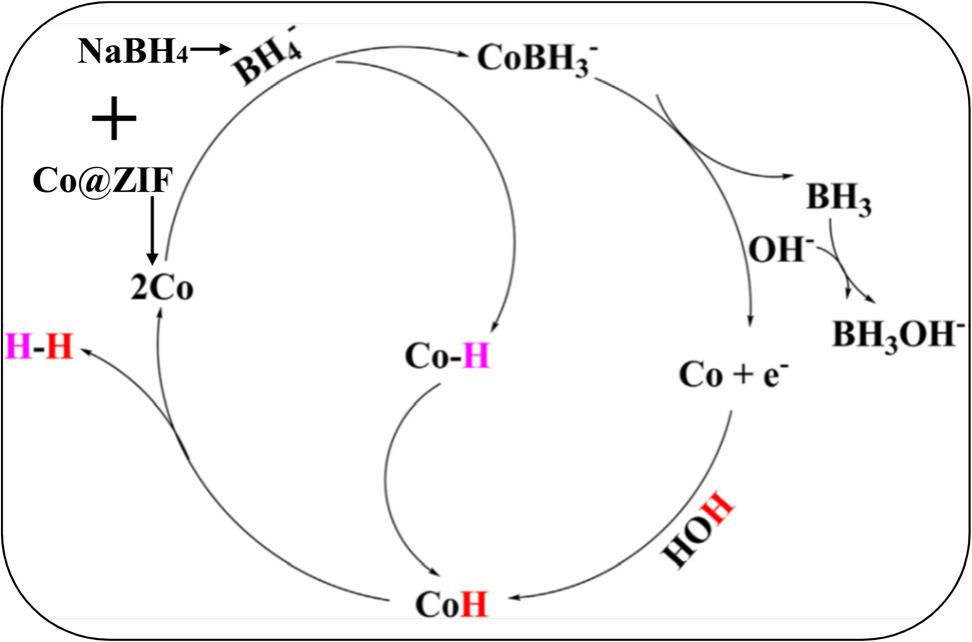
Mechanism involved in the hydrolysis of NaBH_4_ in the presence of Co@ZIF.

Using a hydrolysis reaction setup and the water displacement method, hydrogen was produced from the hydrolysis of NaBH_4_ (Scheme 3). A known quantity of NaBH_4_, a catalytic amount of Co@ZIF-1, 2, 3, 4, and 5, as well as a constant volume of water (50 mL) were all introduced independently in the reaction chamber for this experiment. A magnetic stirrer was attached to the reaction chamber (round-bottom flask), which was connected by a pipe. A pipe connected to the water chamber carried the hydrogen gas produced in the reaction chamber. The empty collecting container, which was set on an electronic weighing balance, was connected to another pipe that had been taken out of the water chamber. The hydrogen gas generated in the reaction chamber traveled through the pipe and developed along the reaction course. As a result, the hydrogen generated during hydrolysis was visible when the released H_2_ gas from the reaction chamber displaces an equivalent amount of water in the collecting chamber. The magnetically active catalyst was extracted from the reaction mixture using an external magnetic bar once the reaction was finished. The separated catalyst was utilized for repeated cycles in the hydrolysis process after being regenerated by washing it with water three to four times, and then ethanol, and finally drying it in a hot air oven for 12 h at 90 °C.

**Fig. 6:**
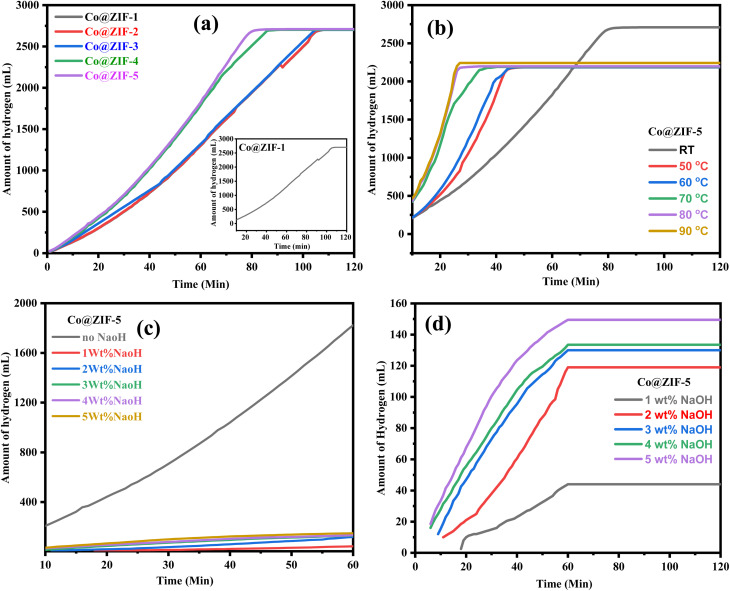
(a) Effect of different Co@ZIF crystals on the hydrolysis rate at room temperature, (b) effect of temperature on hydrolysis by Co@ZIF-5, (c) effect of NaOH on the hydrolysis rate by Co@ZIF-5 and (d) enlarged view of the effect of NaOH on the hydrolysis rate by Co@ZIF-5.

The reaction time directly affects the volume of hydrogen produced, but only up to the point where NaBH_4_ is fully consumed or the catalyst is deactivated. A longer reaction time for synthesis shortens the required reaction time to achieve the maximum hydrogen generation. Optimization of reaction time is crucial for hydrogen generation to ensure efficiency. Fig. 6(a) represents hydrogen generation with different types of ZIF crystals at various reaction times, *i.e.*, different types of ZIF nanocrystals were synthesized at different various reaction times such as 60 min, 90 min, 120 min, 150 min, and 180 min and were named Co@ZIF-1, Co@ZIF-2, Co@ZIF-3, and Co@ZIF-4, Co@ZIF-5 catalysts, respectively. Here, we can observe that the 2701 mL hydrogen is generated in 80 min with ZIF-5, whereas we observed 1910, 1915, 1920, 2480, and 2701 mL in 80 min with Co@ZIF-1, Co@ZIF-2, Co@ZIF-3, Co@ZIF-4 and Co@ZIF-5, respectively. Here, we conclude that the Co@ZIF-5 is the most prominent catalyst for generating hydrogen efficiently.

### 4.2. Effect of reaction temperature

The reaction temperature ranged from room temperature to 90 °C to ascertain how temperature affects hydrolysis. The process of producing hydrogen critically depends on temperature; the higher the temperature, the more hydrogen is produced. For the hydrolysis of NaBH_4_ catalyzed by 0.050 g of Co@ZIF using 1.0 g of NaBH_4_ in 50 mL of DI water, Fig. 6(b) shows the variation in the hydrogen generation graph with respect to various reaction temperatures such as 50 °C, 60 °C, 70 °C, 80 °C, and 90 °C. The amount of hydrogen produced in 80 min at room temperature was measured to be 2701 mL. However, when exposed to a higher temperature of 60 °C, the hydrogen generation is high within a shorter time. Still, it was saturated at 2061 mL in 42 min, and it is less than the hydrogen generated at room temperature due to the decomposition of ZIF at this temperature in the presence of NaBH_4_. In this instance, the temperature had a major impact on the reaction rate but showed no noticeable changes in the amount of hydrogen generated. The reaction was finished in 36 min at 70 °C and reached saturation at the same temperature as at 600 °C. The hydrogen generation then further increased at 80 °C and 90 °C, reaching 2198 and 2230 mL, respectively. Similarly, the kinetics of these responses were excessively quick; the response reached saturation in just 28 and 26 min, respectively. Thus, it is shown that the reaction kinetics were improved with respect to temperature by employing a catalytic quantity of Co@ZIF crystals when the reaction temperature was increased from room temperature to 90 °C. Accordingly, the results show that compared to the higher temperatures, as shown in Fig. 6(b), the hydrolysis of NaBH_4_ carried out at ambient temperature produced the most effective results. Consequently, we believe that the ideal temperature is room temperature with green chemistry and lower energy use.^44^

### 4.3. Effect of basicity (NaOH) on hydrolysis rate

By altering the NaOH concentration between 1–5 wt% in relation to the weight of NaBH_4_, the impact of basicity on the hydrolysis of NaBH_4_ was examined for the Co@ZIF-5 catalyst. Given that adding a base, such NaOH, can prevent NaBH_4_ from hydrolyzing spontaneously, alkaline-stabilized NaBH_4_ solution is always utilized as the feedstock for hydrogen generation. However, the dynamic behavior of NaBH_4_ hydrolysis during hydrogen generation might be affected by the presence of NaOH. Fig. 6(c) illustrates how the concentration of NaOH affected the generation of hydrogen for the Co@ZIF-5 catalyst. Fig. 6(d) shows that when NaOH was added, the formation of hydrogen changed drastically. NaOH is advantageous for the quick formation of CoB active centers in the ZIF catalyst and can slow down the formation of the active catalytic complex by protonation and coordination in solution, as evidenced by the gradual decrease in the initial hydrogen production rate with an increase in NaOH concentration. ZIF framework complexes exhibit a comparable activating action, although the de-protonation of ZIF cannot be enhanced. Compared to hydrogen produced without NaOH, they produce insufficient amounts of hydrogen, according to the intermediate reaction process. In conclusion, NaOH has a significant impact on the pace of hydrogen generation and can only affect the initial formation of CoB. However, due to its caustic nature, it can degrade the generation of hydrogen, and it can lead to the formation of excess byproducts, which decrease the rate.^25^

### 4.4. Effect of acidity (HCL) on hydrolysis rate

**Fig. 7:**
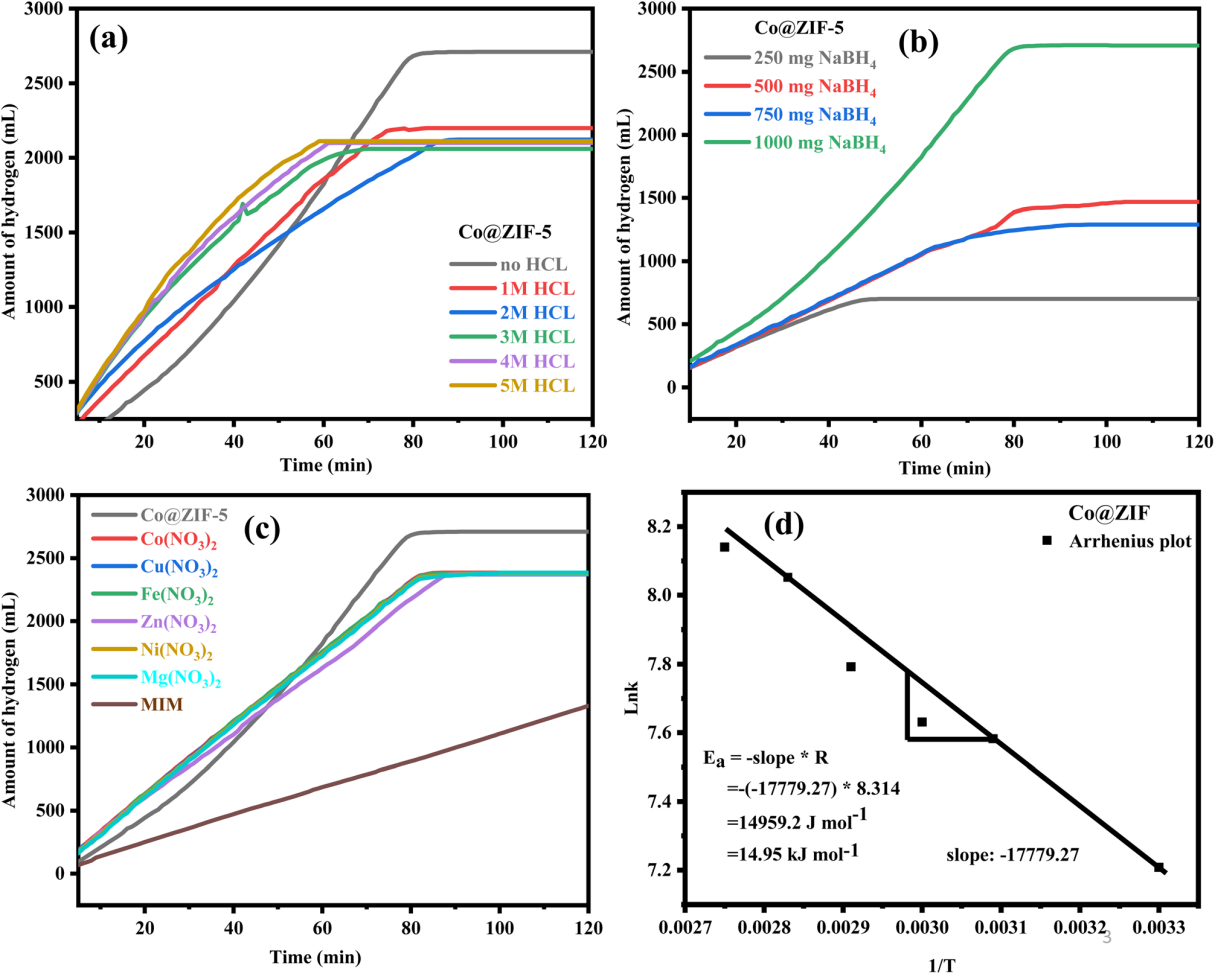
(a) Effect of HCl on hydrolysis by Co@ZIF-5, (b) effect of NaBH_4_ concentration on hydrolysis by the Co@ZIF-5 catalyst, (c) effect of homogeneous catalysts on the hydrolysis of NaBH_4_, and (d) Arrhenius plot.

The effect of acidity on the hydrolysis of NaBH_4_ of the as-synthesized Co@ZIF-5 catalyst was examined by changing the HCl concentration from 1 M to 5 M with respect to the weight of NaBH_4_. The acid concentration plays a vital role in the hydrogen generation process; a higher molar concentration of HCl increases hydrogen generation. The volume of hydrogen generated was evaluated to be 2701 mL in 80 min without HCl. However, the hydrogen generation was high within a shorter time when subjected to a molar concentration of 1 M of HCl, which became saturated at 2190 mL in 77 min. This is less than the hydrogen generated at room temperature due to the decomposition of Co@ZIF at the reaction temperature in the presence of NaBH_4_ for the Co@ZIF-5 catalyst,^45^ as shown in Fig. 7(a). At 2 M, the reaction was accomplished in a reaction time of 70 min, and similarly, became saturated at the same point where it occurs at 2 M. Subsequently, with 4 M and 5 M, the hydrogen generated was 2054 and 2058 mL, individually. Likewise, in these reactions, the response kinetics was very quick; the reaction was finished in 60 and 56 min, respectively, and reached saturation.

### 4.5. Effect of reducing agent concentration

We also measured the effects of NaBH_4_ on the hydrolysis process. A series of tests was conducted for the Co@ZIF-5 catalyst using various NaBH_4_ concentrations, including 0.25 g, 0.50, 0.75, and 1.0 g in 50 mL DI water, to evaluate the effect of NaBH_4_ concentration on the volume of hydrogen generated at room temperature. The results for a variation in NaBH_4_ concentration on the Co@ZIF-5 catalyst are shown in Fig. 7(b). The hydrogen generation rate increased nearly linearly from 701 mL to 2701 mL as the concentration of NaBH_4_ increased from 0.25 to 1.0 g. However, taking into account atom economy, a weight of 1.0 g was chosen as the ideal concentration of NaBH_4_ given that it produced 2701 mL of hydrogen by fully utilizing NaBH_4_ and had a very high yield of hydrogen. However, in this instance, the results show that faster comparative kinetics is achieved with a lower NaBH_4_ concentration than with a high NaBH_4_ concentration. This can be explained by the decrease in mass transport, where the mass transfer and heat transfer effects have an incompatible rate dependence. This influences the rate of hydrogen generation by allowing more NaBH_4_ and water to come into contact with the catalytic surface.^44^

### 4.6. Effect of homogeneous catalysts

The effect of some homogeneous catalysts on the hydrogen generation with the as-synthesized Co@ZIF-5 catalyst is presented in Fig. 7(c). According to this graph, we can observe some of the effects of homogeneous catalysts such as Co(NO_3_)_2_, Cu(NO_3_)_2_, Fe(NO_3_)_2_, Zn(NO_3_)_2_, Ni(NO_3_)_2_, and Mg(NO_3_)_2_ on the hydrolysis of NaBH_4_. Here, the above-listed homogeneous catalysts generate the same amount of hydrogen, except MIM (2-methyl imidazole), but the Co@ZIF-5 catalyst showed a higher H_2_ generation rate. The rate of hydrogen generation by the Co@ZIF-5 catalyst among various homogeneous catalysts is presented in Fig. 7(c). We conclude that homogeneous catalysts also show the maximum hydrogen generation along with the Co@ZIF catalyst. Homogeneous catalysts significantly enhance the hydrogen generation rate and efficiency during NaBH_4_ hydrolysis but pose challenges in terms of recovery and reusability. However, we had chosen Co@ZIF-5 as a prominent catalyst in hydrogen generation due to the lack of catalyst regeneration in the homogeneous phase after the hydrolysis of NaBH_4_.

### 4.7. Evaluation of activation energy & reaction rate for Co@ZIF-5 catalyst

The reaction rate and activation energy were calculated to determine the catalyst activity, and the preliminary hydrogen generation rate *versus* temperature was used to define the activation energy of the Co@ZIF-catalyzed hydrolysis reaction. Fig. 7(d) shows the Arrhenius plot of ln(*K*) *versus* 1/*T*.

According to the Arrhenius plot,

2 *K* = *A* − *e*^−*E*_*α*_/*RT*^

where *K* = hydrogen generation rate (L min^−1^ g^−1^), *A* = pre-exponential factor, *E*_*α*_ = activation energy (kJ mol^−1^), *R* = gas constant (8.314 J mol^−1^), and *T* = solution temperature (K).

#### 4.7.1. Calculation of hydrogen rate

The volume of generation of hydrogen in the hydrolysis of NaBH_4_ in the presence of Co@ZIF was found to be 2701 mL in 80 min. Now, the hydrogen generation rate (K) is calculated by multiplying the amount of hydrogen generated by half the time, as shown in eqn (3)–(6).3
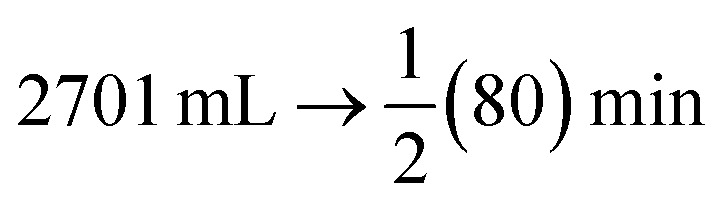

4 *X* mL → 1 min

Here, we generated 67.525 mL of hydrogen in a minute.

We calculated the hydrogen generation rate with 1000 mg of catalyst in the units of g mL^−1^ min^−1^.

5 67.525 mL → 0.05 g

6 *X* mL → 1.0 g

After the calculations, we analyzed the hydrogen generation rate of 1350.5 mL min^−1^ g^−1^.

The activation energy of Co@ZIF-catalyzed NaBH_4_ hydrolysis was determined to be 14.95 kJ mol^−1^ based on the slope of the line. According to the slope of the line, the Co@ZIF catalyst activation energy (14.95 kJ mol^−1^) for the hydrolysis of NaBH_4_ demonstrated a first-order reaction in water as the solvent, which coordinated the particular concentration of NaBH_4_ with the Co@ZIF catalyst. An example of the Arrhenius plot is Fig. 7(d). The prepared catalyst is a highly effective catalyst for hydrogen generation *via* the hydrolysis of NaBH_4_, as indicated by the Arrhenius equation, which states that a drop in activation energy will result in an exponential increase in rate *K* and low *E*_a_.^46^ The results indicate that the Co@ZIF catalyst has significantly higher catalytic activity (2701 mL) and lower activation energy (14.95 kJ mol^−1^) at room temperature. A comparison of the obtained results for the Co@ZIF-5 catalyst in hydrogenation among earlier reported Co-based catalysts is shown in Table 2.

**Table 2:** Comparison of conditions, HGR and *E*_a_ values of the as-synthesized Co@ZIF-5 catalyst for the hydrolysis of NaBH_4_ among earlier reported cobalt-based catalysts

Sr. no.	Catalyst	Conditions	HGR (mL min^−1^ g^−1^)	*E*_a_ (kJ mol^−1^)	Ref.
1	Co/AC1	1 wt% NaOH, 5 wt% NaBH_4_, 250 mg catalyst, 30 °C	1280	—	47
2	Co–B/HPCM	2.25 wt% NaOH, 2 wt% NaBH_4_, 100 mg catalyst, 25 °C	3083.1	43.3	48
3	Co@C	2 wt% NaOH, 5 wt% NaBH_4_, 20 mg catalyst, 30 °C	1680	45	49
4	Co/TiO_2_	3.7 wt% NaOH, 3.7 wt% NaBH_4_, 50 mg catalyst, 25 °C	660	45.2	50
5	Co@X-TA-C	1 wt% NaOH, 1 wt% NaBH_4_, 30 mg catalyst, 25 °C	100.7	15.2	51
6	Co–O–P	8 wt% NaOH, 4 wt% NaBH_4_, 10 mg catalyst, 25 °C	4850	—	52
7	Co@C	NaOH (0.1 g), NaBH_4_ (0.1 g), 100 mg catalyst, 25 °C	5392	32.7	47
8	NiCo/ZIF-67/rGO	0 wt% NaOH, NaBH_4_ (85 mg), 50 mg catalyst, 303 K	5420	21.08	5
9	Co-ZIF-9	5 wt% NaOH, NaBH_4_ (0.5 wt%), 25 mg catalyst, 40 °C	3641	—	9
10	Cobalt-ZIF	0 wt% NaOH, 200 mg NaBH_4,_ 15 mg catalyst (35 °C)	720.7	63.74	15
11	Co-ZIF-8	0.4 mol L^−1^ NaOH, 0.75 mmol NaBH_4_, 4 mmol% catalyst (30 °C)	14 023	62.9	16
**12**	**Co@ZIF-5**	**0 wt% NaOH, 1 g NaBH**_**4**_ **, 50 mg catalyst, RT (25 °C)**	**1350.5**	**14.95**	**This work**

## 5. Evaluation and validation of results obtained for Co@ZIF catalyst in hydrolysis reaction *via* theoretical study

### 5.1. Validation of structural and micro-structural properties of Co@ZIF-5

**Fig. 8:**
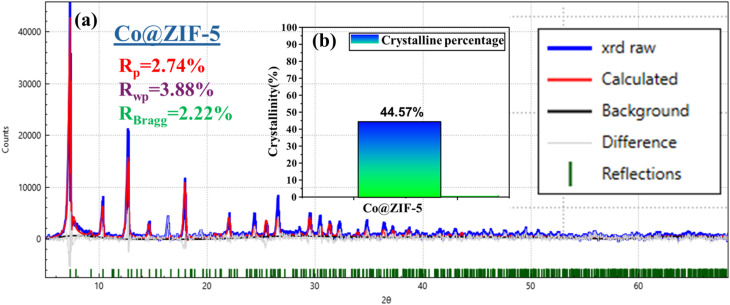
XRD refinement of (a) Co@ZIF-5 and (b) crystalline percentage histogram of the prepared sample.

According to the refinement results, we learned that before being employed in the hydrolysis of sodium borohydride, the Co@ZIF catalyst crystallized in the hexagonal *P*6_3_ space group. Its structure consisted of six NH_4_ clusters within a CoH_3_(CO_2_)_3_ framework, where the Co^2+^ atoms exhibited octahedral coordination with oxygen atoms. The Co–O bond length of the catalyst was 4.316 Å, contributing to the its structural integrity. The calculated Rietveld refinement of the synthesized fresh Co@ZIF-5 catalyst is presented in Fig. 8(a), respectively. As presented in Fig. 8(b), the percentage crystallinity of the fresh synthesized Co@ZIF-5 catalyst is 44.57%, which can be attributed to its structural rearrangements and reorganization during the catalytic reaction. Co@ZIF likely contains a mixture of amorphous and crystalline regions in its original state.

**Fig. 9:**
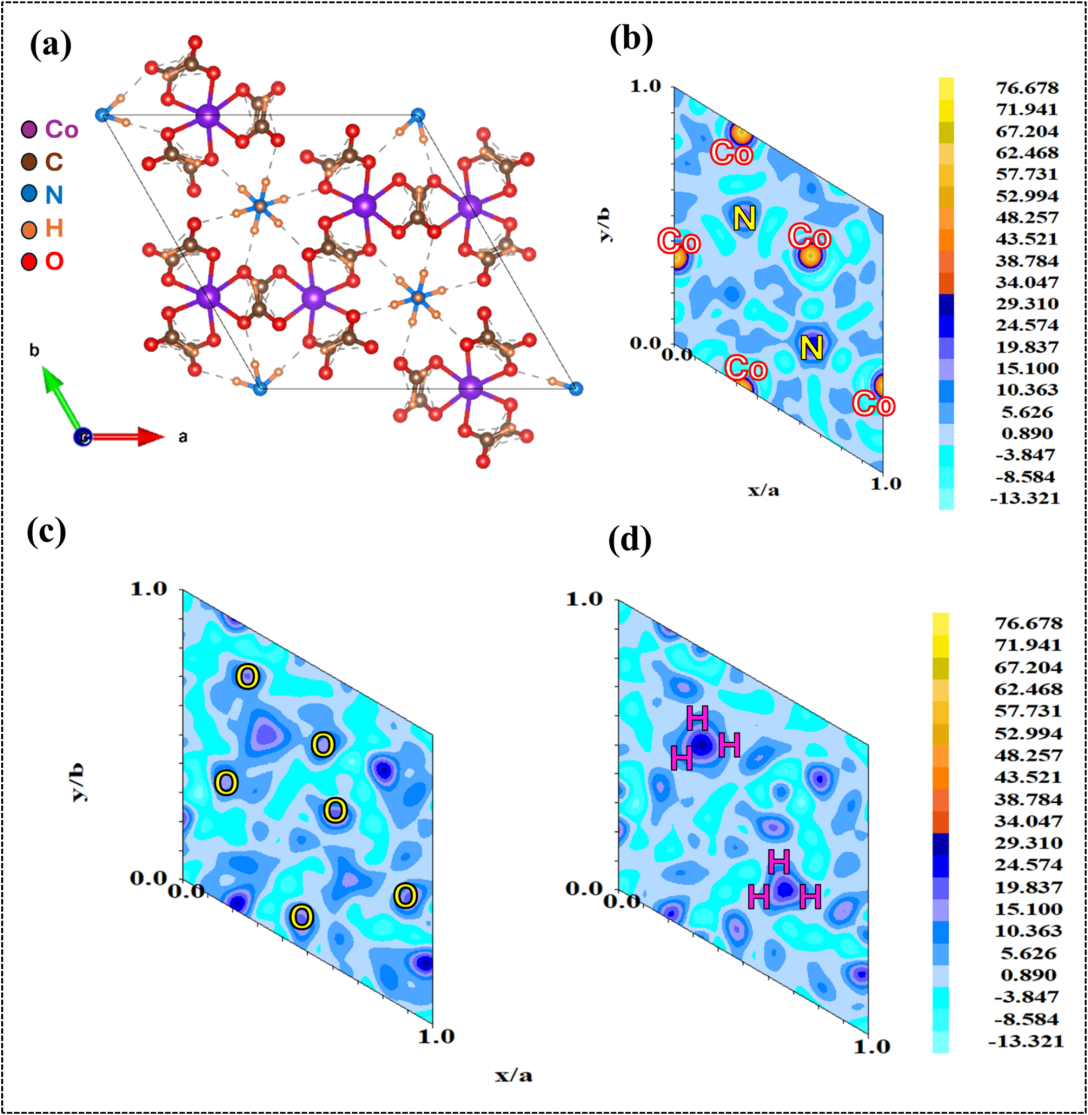
(a) Structure and (b–d) electron density mapping of the Co@ZIF-5 catalyst.

Following the Rietveld refinement of the XRD pattern of the Co@ZIF-5 catalyst, the structure of the cobalt-doped ZIF catalyst was constructed using the VESTA software. The generated crystal structure of the Co@ZIF-5 catalyst is shown in Fig. 9(a). Subsequently, electron density mapping was carried out using the FullProf software to provide insights into the atomic positions and potential changes in the bonding environment during catalysis. The electron density mapping revealed distinct changes in the density distribution around the Co^2+^ atoms before and after the hydrolysis application. This mapping also highlights which parts of the catalyst framework remain stable. Electron density mapping has proven to be an invaluable tool in identifying the active sites and understanding the atomic-level changes.^53^ These insights into electron-density redistribution provide a deeper understanding of the performance and structural stability of the catalyst. The electron density of the fresh catalyst is shown in Fig. 9(b–d).

Another contributing factor is the release of strain within the Co@ZIF-5 catalyst, where the material may have been under microstrain, contributing to its disordered structure. The crystallite size of Co@ZIF-5 is 65.70 nm, which plays a role in this improvement in crystallinity. The unit cell volume of Co@ZIF-5 is 2619.08 Å^3^, which suggests atomic-level reorganization, resulting in more atoms aligning into a regular, repeating lattice pattern. The freshly synthesized catalyst has a hexagonal phase, also supporting this structural reorganization. The electron density mapping reveals the redistribution of electron density around the cobalt atoms, indicating that the atomic bonds reorganize to form a more crystalline structure.

The parameters provided in Table 3 for the Co@ZIF-5 catalyst before hydrolysis give insights into its structural and physical properties, which are crucial for understanding its catalytic performance. The *a* and *c* lattice parameters were obtained using the Rietveld refinement results, which are 28.08 nm and 18.47 nm for Co@ZIF-5, respectively. This suggests that Co@ZIF-5 has a more complex or expanded structure, potentially leading to enhanced catalytic sites and activity. The data for the number of unit cells per crystallite (*N*_u_), unit cell volume (*v*), stress (*σ*), crystallite volume (*V*), microstrain (*ε*), and dislocation density for the Co@ZIF-5 catalyst are shown in Table 3. The volume of the unit cell is 2619.08 Å^3^.^54^

**Table 3:** Microstructural properties of Co@ZIF-5 catalyst

Sr. no.	Parameters	Co@ZIF-5 (before hydrolysis)
1	Lattice parameter (*a*) (nm)	28.08
2	Lattice parameter (*c*) (nm)	18.47
3	Unit cell volume (*v*) (Å)^3^	2619.08
4	Interplanar spacing *d* (Å)	12.1069
5	Average crystallite size (*D*) (nm)	65.70
6	Crystallite volume (*V*_c_) (Å)^3^	283.7125
7	Unit cell volume per crystallite (*N*_u_)	0.108325
8	Micro strain (×10^−3^) (*ε*)	0.5216
9	Stress (*σ*)	−1.7964
10	Dislocation density (*γ*) (10^15^ m^−2^)	0.2316
11	X-ray density (*D*_*x*_) (g cm^−3^)	1.4046
12	Specific surface area (*ρ*) (cm^2^ g^−1^)	65.00837
13	Number of unit cells (n*) ×10^3^	56.69
14	Bond length (*l*) (Å)	4.316

The crystallite size (*D*) and interplanar distance (*d*) of both the samples were calculated using the Debye–Scherrer equation and Bragg's law. The Scherrer equation is given as follows:^55^7
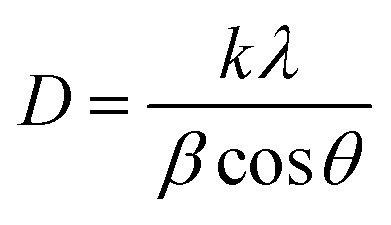
where *K* is the Scherer constant, *λ* = wavelength of the X-ray source, *β* = FWHM, and *θ* is the peak position. Similarly, Bragg's law relates the angle of incidence and diffraction to the X-ray wavelength and the distance between crystal planes.^56^ The equation is expressed as follows:

8 *nλ* = 2*d* sin *θ*

where “*n*” represents the diffraction order, expressed as an integer. “*λ*” denotes the X-ray wavelength. At the same time, “*d*” refers to the interplanar spacing, which indicates the distance between crystal planes. Finally, theta (*θ*) signifies the angle of diffraction. In this study, these equations are fundamental in analysing the crystallinity and structural properties of materials.

The interplanar spacing of Co@ZIF-5 is 12.1069 Å, indicating the distance between layers in the crystal structure, which can affect the diffusion of reactants and products during catalysis. The Co@ZIF catalyst has a crystallite size of 65.70 nm.

The microstrain (*ε*) and stress (*σ*) of both samples can be determined using the following relation:9
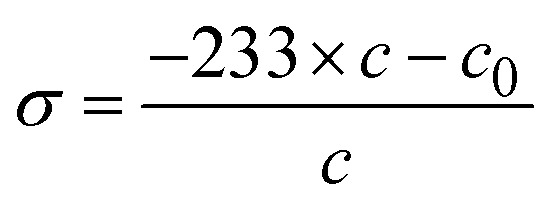
10
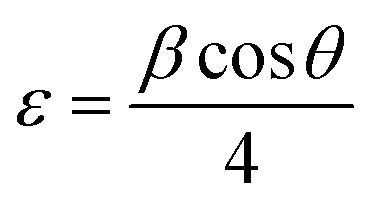


In eqn (10), *c*_0_ signifies the standard lattice parameter from the JCPDS card data, and *c* denotes the lattice parameter obtained from the prepared sample. The microstrain and stress of Co@ZIF-5 (as shown in the table) at in the range of 0.5216 × 10^−3^ and −1.7964, respectively. The microstrain of 0.5216 × 10^−3^ for Co@ZIF indicates less distortion in its crystal structure and lower strain can enhance the stability and performance of the catalyst under the operational conditions.

The dislocation density (*γ*), which can be used to estimate any defect coming from variations in concentration within the sample, can also be used to express the defect concentration in the samples. The formula for dislocation density can be determined using the average crystalline size (*D*), which is given as follows:11
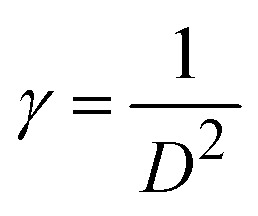


The table shows that the dislocation density is in the range of 0.2316 × 10^15^ m^−2^. The number of unit cells (n*) in Co@ZIF-5 could be calculated based on the following relation.12
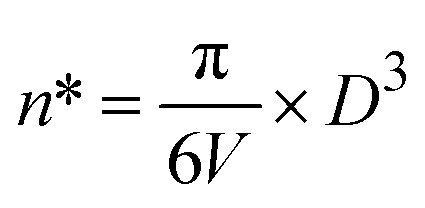


The table shows that the number of unit cells per crystallite is 0.108325, and the bond length is 4.316 Å. The bond length of the catalyst was obtained from the refinement results (as shown in the table), indicating stronger interactions within the structure, which can influence the stability and reactivity of the catalyst. The relationship shown below in eqn (13) was used to evaluate the specific surface area. The specific surface area (as shown in Table 3) of Co@ZIF-5 is 65.008 cm^2^ g^−1^. We calculated the X-ray density (*D*_*x*_) of the prepared sample using the formula shown below in eqn (14).13
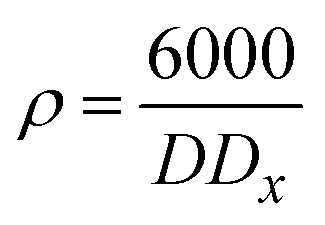
14
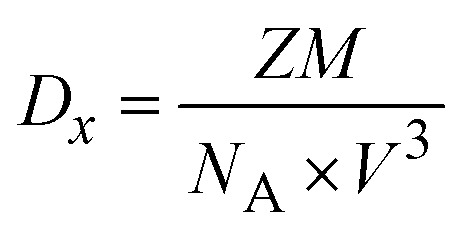
where *Z* denotes the number of molecules per unit cell, *M* the molecular weight, *V* the unit cell volume (all of which were derived from the refinement findings), and *N*_A_ Avogadro's number (6.023 × 10^23^). The X-ray density is 1.4046 g cm^−3^, as shown in Table 3. This suggests that the overall packing density of the atoms within the crystal decreases, possibly due to structural changes or the formation of voids within the framework during the reaction, which we can see through FE-SEM morphology changes. The prepared catalyst exhibits an excellent catalytic performance for hydrogen generation through the hydrolysis of NaBH_4_. The insights from each parameter reflect how the reaction environment causes structural breakdown and reorganization, influencing the hydrogen generation efficiency by the catalyst. Its structural integrity, stability, and recyclability make it a promising candidate for sustainable hydrogen generation, supported by thorough characterization and analysis of its properties. The calculated values from the microstructural analyses of the Co@ZIF-5 catalyst are presented in Table 3.

### 5.2. Determination of elastic properties

The elastic properties of the Co@ZIF-5 sample reveal several significant changes in its structural and bonding characteristics. Table 4 presents the elastic properties derived from the FT-IR spectra and various calculations. These properties help us understand the mechanical stability and flexibility of the catalyst.

**Table 4:** Elastic properties of Co@ZIF-5 catalyst

Sr. no.	Parameters	Co@ZIF-5 (before hydrolysis)
1	Debye temperature (θD*) K	8.0160
2	The effective mass of the M–O bond	12.5830
3	Wavenumber (nm)	688
4	Force constant *k* (×10^29^)	2.1140
5	Bond length of M–O bond (×10^−10^)	4.3162
6	Elastic stiffness constant (*C*_11_) (×10^29^)	0.0752
7	Longitudinal wave velocity (×10^14^)	0.7320
8	Transverse wave velocity (×10^14^)	0.4226
9	Mean elastic wave velocity (×10^14^)	0.4692
10	Rigidity modulus (×10^29^)	0.0250
11	Bulk modulus (×10^29^)	0.0752
12	Youngs modulus (×10^29^)	0.0677
13	Poisson's ratio	0.35
14	Lattice energy (×10^26^)	−1.5161

The bond length of the M–O bond was determined using the unidimensional harmonic oscillator model for infrared absorption analysis.^57^ The following equation shows the relationship between the force constant and wavenumber:15
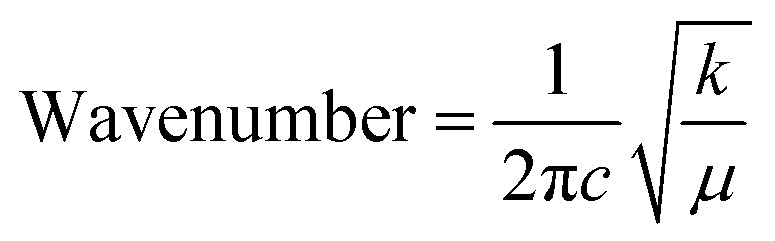
where *k* represents the force constant, *c* the speed of light (3.00 × 10^10^ cm s^−1^), and *μ* the effective mass of the M–O bond given by eqn (16):^58^16
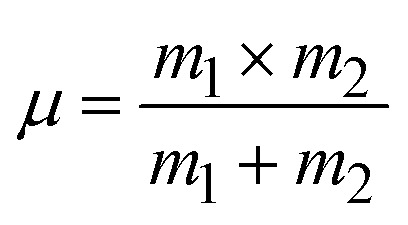
where *m*_1_ and *m*_2_ represent the atomic weights of the bound atoms. The calculated effective masses and force constants of the synthesized catalysts are shown in Table 4, which well matched the XRD results. The observed effective mass of Co@ZIF-5 is 12.5830.

Similarly, at the wavenumber of 688 nm, and the force constant and bond length of the M–O bond are in the range of 2.1140 × 10^29^ and 4.3162 × 10^−10^, signifying closer atomic packing and stronger M–O interactions, respectively. This bond suggests enhanced structural integrity and bonding strength, likely contributing to improved rigidity and stability. The bond stiffness within the lattice atoms caused by the cation redistribution inside the interstitial sites is revealed by the elastic constant. Measuring different elastic moduli, such as elastic stiffness constants, elastic modulus, and elastic wave velocity, allows one to express the elastic nature. The elastic stiffness constant (*C*_11_) was also computed using the following formula.^59^17
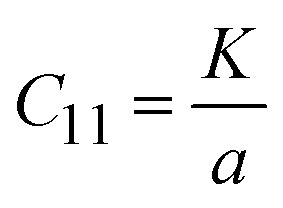


For the as-synthesized catalysts, their elastic stiffness constant (*C*_11_) and Young's modulus are significantly higher before and after hydrolysis. The stiffness constant is in the range of 0.0752 × 10^29^. This mechanical strength is crucial for maintaining the integrity of the active sites. The rigidity modulus, bulk modulus, and Young's modulus of the catalyst are also deliberated by using eqn (18) to (20),^59^ as follows:

18 Rigidity modulus (*R*) = *D*_*x*_*V*_t_^2^

19
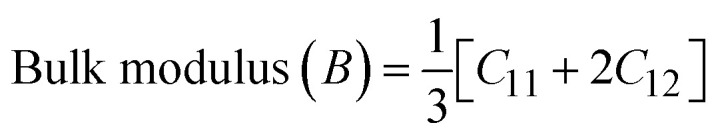
20
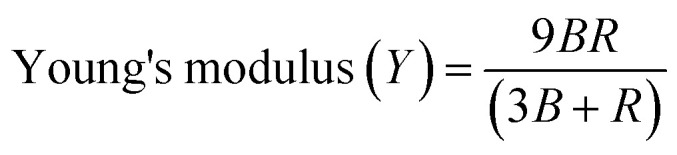


The Poisson's ratio of the prepared samples was calculated as follows.^36^21
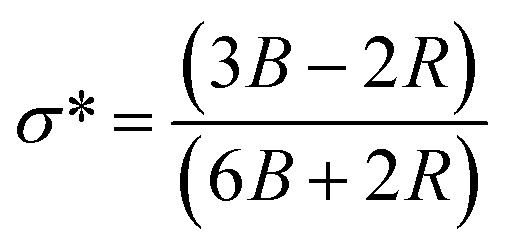


Based on the theory of elasticity, the values of σ* often fall between 1–0.5. The Poisson's ratio (*σ*) is consistent at 0.35, implying that the stiffness and elasticity of the material changed its volumetric response to stress (ratio of transverse to longitudinal strain).

The longitudinal (*V*_l_) and transverse (*V*_t_) elastic wave velocities were calculated using the elastic stiffness constant and given in Table 4.^60^ The wave velocities suggest that the material can withstand thermal and mechanical stress, making it ideal for catalytic use without degradation. This enhanced stability, coupled with the bi-functional acid–base properties, improves the performance of Co@ZIF-5 by maintaining the availability of its active sites and mechanical integrity over catalytic cycles. Compared to the transverse elastic wave, the longitudinal elastic wave travels faster. As the wave travels through the medium, energy is transferred to each particle, causing the particle to vibrate. Using the energy from the vibrating particle, another particle in the medium also vibrates. The particle in the medium vibrates in the direction of the longitudinal elastic waves. Consequently, less energy is needed for the surrounding particles to vibrate and the energy of the waves is greatly boosted. Therefore, the longitudinal wave velocity is more significant. The mean elastic wave velocity (*V*_m_) can be expressed as follows:^61^22
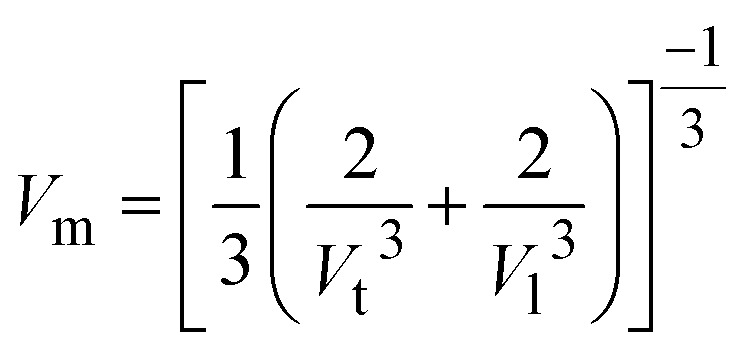


The Debye temperature ranges 8.0160 K. The lattice energy can be used to explain the strength of the bonds inside the catalyst. Lattice energy is the potential energy created when atomic orbitals traverse in a crystal structure. It is calculated using eqn (23).^58^

23 *U*_L_ = −3.108 (*MV*_m_^2^) × 10^−5^ eV

where *M* denotes the molecular weight of the sample and *V*_m_ represents the mean elastic wave velocity. Lattice energy is released when ions in a crystal lattice come together from infinity. It measures the strength of the ionic bonds in a solid. It dictates the stability of the metal–oxygen lattice and the strength of the ionic bonds. The lattice energy in the range of −1.5161 × 10^26^ suggests that the structure becomes more stable and energetically favourable for hydrolysis, which is likely due to the reorganization and strengthening of bonds within the framework.

### 5.3. Catalyst recyclability study

**Fig. 10:**
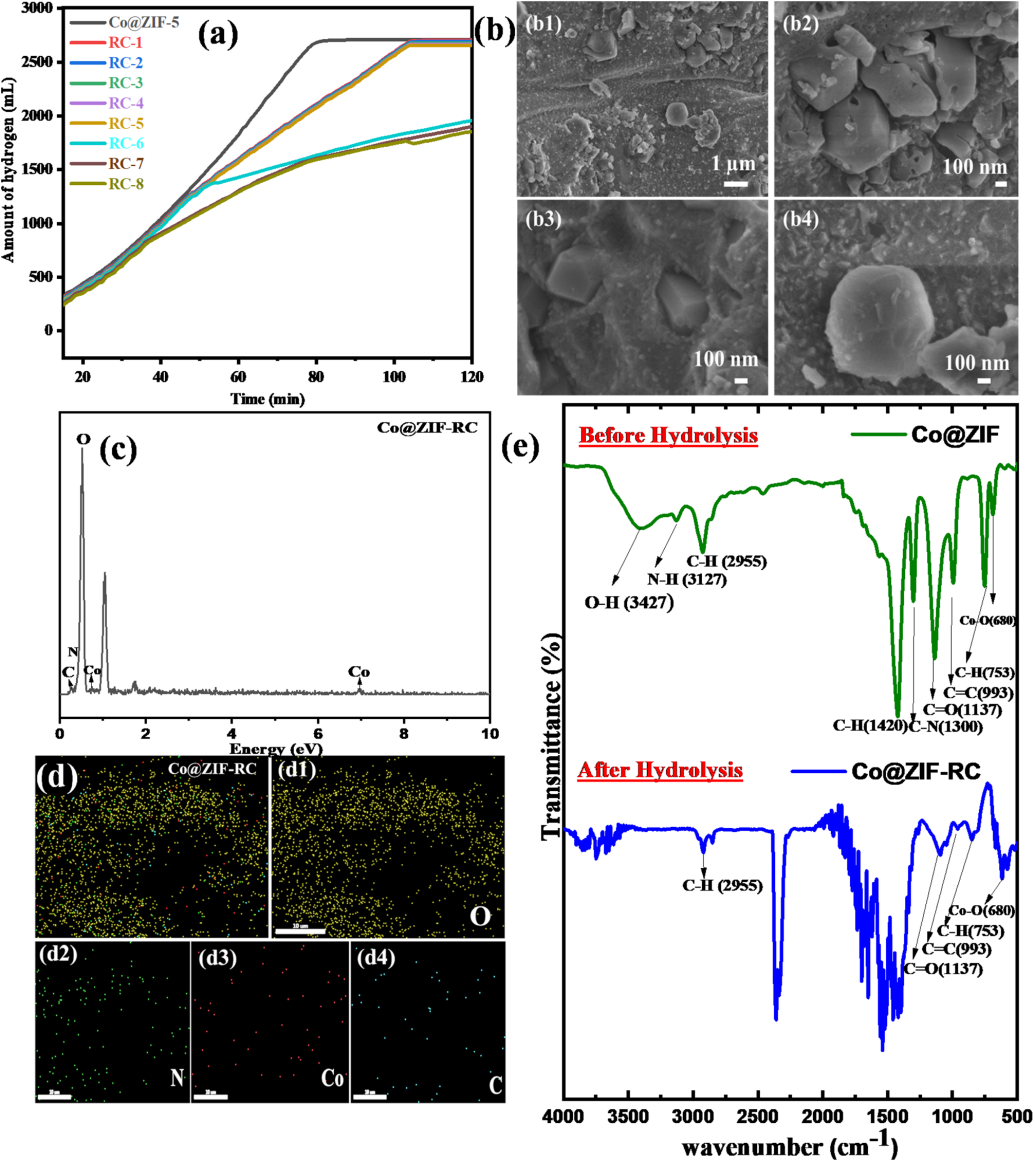
(a) Recyclability of the catalyst in repeated hydrolysis cycles, (b) FE-SEM images of the recycled Co@ZIF-5 catalyst, (c) EDX spectra of Co@ZIF-RC nanocrystals, (d), (d1–d4) elemental mapping of the Co@ZIF-RC catalyst, and (e) FT-IR spectra of the Co@ZIF-5 and Co@ZIF-RC catalysts.

Studies on recycling are essential in the realm of heterogeneous catalysis. The recycling ability of Co@ZIF for hydrolyzing NaBH_4_ was assessed; the results are displayed in Fig. 10(a). The catalyst was separated using an external magnet once the reaction was finished, and the Co@ZIF catalyst was rinsed 3–4 times with ethanol and water for the recycling procedure. Then, the resulting slurry was dried for 12 h at 90 °C in a hot air oven. Under the ideal reaction conditions, the catalytic activity findings of the repurposed catalyst for up to five cycles are displayed in Fig. 10(a). According to the observations, the Co@ZIF catalyst maintained a comparable hydrogen generation yield with negligible loss. However, following the second cycle, a slight drop-in catalytic activity was noted. This could be because sodium metaborate, a by-product of each reaction cycle, collected on the catalyst surface and was difficult to remove with a simple water wash. As a result, 2701 mL of hydrogen was produced by the fresh catalyst, and similar findings were obtained during the second and third cycles (2190 mL and 2180 mL, respectively). In contrast, the fourth and fifth cycles demonstrated a total hydrogen generation of 2170 mL and 2159 mL in an 80 min reaction time, respectively. Afterward, from 6 to 10 cycles, the hydrogen generation was too low compared with the first five cycles. During the use of the catalyst, its activity is reduced because of the gradual formation of Co–B deposits on its surface. The regeneration temperature and time were well adjusted to prevent the catalyst from sintering. During the operation of the Co-based catalyst, the catalyst loses its activity due to more borate deposits (Co–B) on its surface. In this type of catalyst, its activity can be restored to a normal level through *in situ* technology, but during the regeneration process, it is easier to cause the performance of the catalyst to change. We conclude that the Co@ZIF-5 catalyst has an excellent yield of hydrogen generation during the hydrolysis process and can be recycled effectively for up to five cycles.

We analyzed the recycled Co@ZIF-RC catalyst using various analytical techniques for the confirmation of its structural and morphological changes. Therefore, the morphological aspects of the recycled catalyst were examined using FE-SEM, EDX, elemental mapping, and FT-IR analyses. The surface morphology of the recycled Co@ZIF was examined by FE-SEM, as shown in Fig. 10(b), which does not show the morphology of fresh catalyst due to the heavy accumulation of sodium metaborate on the catalyst, but still the recycled catalyst maintained some hexagonal nanocrystals. The EDX report for the recycled Co@ZIF showed the presence of Co, C, N, O, and Na elements. The EDX analysis reveals the presence of additional Na metal due to the formation of a byproduct in sodium borohydride hydrolysis. The new peaks associated with sodium and oxygen exhibited a higher intensity, as shown in Fig. 10(c). Even though oxygen was present in Co@ZIF, its oxygen content was significantly augmented due to NaBO_2_ and H_2_O. The elemental composition of the Co@ZIF-RC catalyst is shown in Fig. 10(d), showing the well dispersion of the elements present in the catalyst. The elemental mapping of the elements present in the Co@ZIF-RC catalyst is shown in Fig. 10(d1–d4).^28^ The FT-IR analyses of the Co@ZIF-5 and Co@ZIF-RC catalysts are shown in Fig. 10(e). This comparison elucidates the structural and chemical changes due to the hydrolysis of Co@ZIF-5. The loss or weakening of O–H, N–H, and other characteristic peaks and the appearance or enhancement of additional peaks suggest the decomposition of the ZIF structure, involving metal–ligand bond disruption, framework collapse, or formation of new bonds.^24^

### 5.4. Plausible mechanism of Co@ZIF catalyst for hydrolysis of NaBH_4_

A plausible mechanism for the hydrolysis of NaBH_4_ with the Co@ZIF catalyst was proposed for the in-depth analysis of the interaction of the catalyst with NaBH_4_. The reaction involved is shown in eqn (24). The mechanism involved in the hydrolysis of NaBH_4_ in the presence of Co@ZIF is represented below in Scheme 3.

24 NaBH_4_ + 2H_2_O → NaBO_2_ + 4H_2_

The hydrolysis of NaBH_4_ with the Co@ZIF catalyst for hydrogen generation involves various steps. Firstly, NaBH_4_ interacts with the cobalt catalyst and NaBH_4_ dissociates in water to release the BH_4_^−^ anion and forms the cobalt borohydride intermediate (CoBH_3_). BH_4_^−^ approaches the catalyst surface (such as a cobalt site in Co@ZIF). The metal centre eases the cleavage of the B–H bonds. This step involves the coordination of the BH_4_^−^ ion with cobalt atoms. CoBH_3_ breaks down to release BH_3_ and cobalt hydride (Co–H). BH_3_ reacts with hydroxide ions (OH^−^) from water to form BH_3_OH^−^, progressing toward borate species (*e.g.*, BO_2_^−^), a known product of hydrolysis. The generation of BH_3_ and regeneration of Co are accompanied by electron transfer, indicating that the catalyst undergoes redox cycling. Co–H undergoes further reaction with water to produce H_2_ and regenerate Co, completing the cycle. Water facilitates this proton transfer step, forming H_2_ through Co–H and proton interaction. This scheme effectively draws the hydrolysis of NaBH_4_ into the catalytic cycle, highlighting the key role of Co in hydrogen generation through the formation and consumption of the hydride intermediate. The hydride (H^−^) ions from BH_4_^−^ are protonated by water. After complete hydrogen release, BH_4_^−^ is oxidized to metaborate (BO_2_^−^). The continuous regeneration of active cobalt sites ensures sustained H_2_ release, which is crucial to generate hydrogen.^62^

## 6. Conclusions

This work successfully synthesized heterogeneous Co@ZIF (ZIF-zeolitic imidazolate frameworks) nanocrystal catalysts without using substrates such as rGO and MXene, and evaluated their efficacious catalytic activity in hydrolysing NaBH_4_ to generate hydrogen. The catalyst produced an excellent amount of hydrogen (2701 mL) at a rate of 1350.5 mL min^−1^ g^−1^ in 80 min at room temperature (25 °C) under ambient reaction conditions because of the synergistic effect of the Co@ZIF crystals. Furthermore, the Co@ZIF-catalysed first-order hydrolysis process had the lowest activation energy of 14.95 kJ mol^−1^ because of this synergistic impact. It is important to note that no reports of such low activation energy for a Co@ZIF catalyst have been reported to date. The ideal hydrolysis reaction conditions with the Co@ZIF catalyst are 50 mg of catalyst and 1000 mg of NaBH_4_ in 50 mL of deionized water, according to the prime hydrogen generation procedure evaluated using different reaction parameters at room temperature. As the concentration of the reactant (NaBH_4_) and catalyst (Co@ZIF) increased, the rate of reaction for NaBH_4_ hydrolysis also increased. Without expressively losing its catalytic activity or physiochemical characteristics, the prepared Co@ZIF-5 catalyst could be utilized up to five cycles in the hydrolysis process. The validation of the crystal structure and the hydrolysis results were evaluated with theoretical calculations as well. The primary benefit of this process is its reliable hydrogen generation at room temperature with recyclable and environmentally friendly catalysts. Furthermore, it is important to note that the prepared Co@ZIF-5 catalyst had a low activation energy and a high rate of hydrogen generation in the hydrolysis of NaBH_4_. However, its catalytic activity was primarily conducted under controlled laboratory conditions, which may not fully represent the practical operating environments. Also, the long-term stability and recyclability tests, though encouraging, were limited in duration, and thus the possible catalyst deactivation mechanisms (such as leaching and structural degradation) need further in-depth analysis. Moreover, this study did not extensively explore the scalability of the catalyst synthesis, which is a critical factor for real-world applications. Future work can focus on addressing these aspects by evaluating the feasibility of this catalyst under industrially relevant conditions.

## Conflicts of interest

There are no conflicts to declare.

## Data Availability

Data will be made available from the corresponding author upon reasonable request.
